# Strategic Priming with Multiple Antigens can Yield Memory Cell Phenotypes Optimized for Infection with *Mycobacterium tuberculosis*: A Computational Study

**DOI:** 10.3389/fmicb.2015.01477

**Published:** 2016-01-06

**Authors:** Cordelia Ziraldo, Chang Gong, Denise E. Kirschner, Jennifer J. Linderman

**Affiliations:** ^1^Department of Chemical Engineering, University of Michigan, Ann ArborMI, USA; ^2^Department of Microbiology and Immunology, University of Michigan Medical School, Ann ArborMI, USA; ^3^Department of Computational Medicine and Bioinformatics, University of Michigan, Ann ArborMI, USA

**Keywords:** subunit vaccine, T cell priming, cell-mediated immunity, agent-based model

## Abstract

Lack of an effective vaccine results in 9 million new cases of tuberculosis (TB) every year and 1.8 million deaths worldwide. Although many infants are vaccinated at birth with BCG (an attenuated *M. bovis*), this does not prevent infection or development of TB after childhood. Immune responses necessary for prevention of infection or disease are still unknown, making development of effective vaccines against TB challenging. Several new vaccines are ready for human clinical trials, but these trials are difficult and expensive; especially challenging is determining the appropriate cellular response necessary for protection. The magnitude of an immune response is likely key to generating a successful vaccine. Characteristics such as numbers of central memory (CM) and effector memory (EM) T cells responsive to a diverse set of epitopes are also correlated with protection. Promising vaccines against TB contain mycobacterial subunit antigens (Ag) present during both active and latent infection. We hypothesize that protection against different key immunodominant antigens could require a vaccine that produces different levels of EM and CM for each Ag-specific memory population. We created a computational model to explore EM and CM values, and their ratio, within what we term Memory Design Space. Our model captures events involved in T cell priming within lymph nodes and tracks their circulation through blood to peripheral tissues. We used the model to test whether multiple Ag-specific memory cell populations could be generated with distinct locations within Memory Design Space at a specific time point post vaccination. Boosting can further shift memory populations to memory cell ratios unreachable by initial priming events. By strategically varying antigen load, properties of cellular interactions within the LN, and delivery parameters (e.g., number of boosts) of multi-subunit vaccines, we can generate multiple Ag-specific memory populations that cover a wide range of Memory Design Space. Given a set of desired characteristics for Ag-specific memory populations, we can use our model as a tool to predict vaccine formulations that will generate those populations.

## Introduction

An estimated 9 million new cases of tuberculosis (TB) are reported annually (WHO, 2014). This number could be an underestimate by a factor of almost 2 due to difficulties diagnosing individuals with latent infection and those living in remote areas of developing countries (WHO, 2014). In addition to a clear demand for more practical and reliable diagnostic methods and more effective regimens of chemotherapy, there is a desperate need for a better vaccine for TB. BCG, the only vaccine currently approved for use, is somewhat effective in children, but loses its protective effects as individuals enter adolescence (Colditz et al., 1994; Ottenhoff and Kaufmann, 2012; Pitt et al., 2013; Kaufmann, 2014). Vaccines are a critical component of the effort to stop TB due to the asymptomatic nature of latent infection and that initial symptoms of active diseases are shared by many pulmonary infections. By the time an infected individual was diagnosed and starts treatment, s/he has likely been contagious for several weeks (Mehra et al., 2013; WHO, 2014). An ideal vaccine, therefore, would either prevent an initial infection from taking hold or would prevent existing latent TB infections from progressing to active disease, reducing risk of reactivation (Karp et al., 2015).

Recently a renewed focus on TB has led to several promising vaccine candidates reaching clinical trials. However, their efficacy in humans is difficult to predict because there is much work remaining to identify immunological correlates of protection (Lin et al., 2015). *Mycobacterium tuberculosis* is an intracellular pathogen, and thus protection requires cell-mediated immunity (Seder and Hill, 2000; Woodland, 2004; Sallusto et al., 2010; Chanzu and Ondondo, 2014), i.e., populations of memory T cells that recognize specific TB antigens (Ags). Because individuals infected with HIV are many times more likely to have a latent TB infection reactivate to active disease, CD4+ T cells are strongly implicated in protection (Nunes-Alves et al., 2014). Many of the recently developed vaccines have focused on generating large populations of CD4+ memory T cells capable of mounting a strong Th1 response (Karp et al., 2015), but recent failures of vaccines that provide this type of response (Tameris et al., 2013; Kaufmann, 2014) together with other recent results (Mittrücker et al., 2007; Kagina et al., 2009) suggest that there is more to the story.

In addition to the quantity of T cells that can recognize an infectious agent, their quality is key (Nunes-Alves et al., 2014). In order for a memory T cell to be effective, it must have the right function(s), in the correct location, at the moment it is needed (Hikono et al., 2007). Therefore, it is critical to consider subtypes of memory T cells, both CD4+ and CD8+, and how they are generated. In 2004 Sallusto and Lanzavecchia delineated two subtypes of memory T cells (Sallusto et al., 2004). Central memory (CM) cells are long-lived and circulate through lymph nodes (LNs) while effector memory (EM) cells circulate through blood and peripheral tissues. This division mirrors the two major functional roles of memory T cells: CM cells are primed in LNs and rapidly expand into a large population of effector cells that quickly respond to infection, while EM cells are available to recognize and act against invading pathogens at peripheral sites.

**FIGURE 1:**
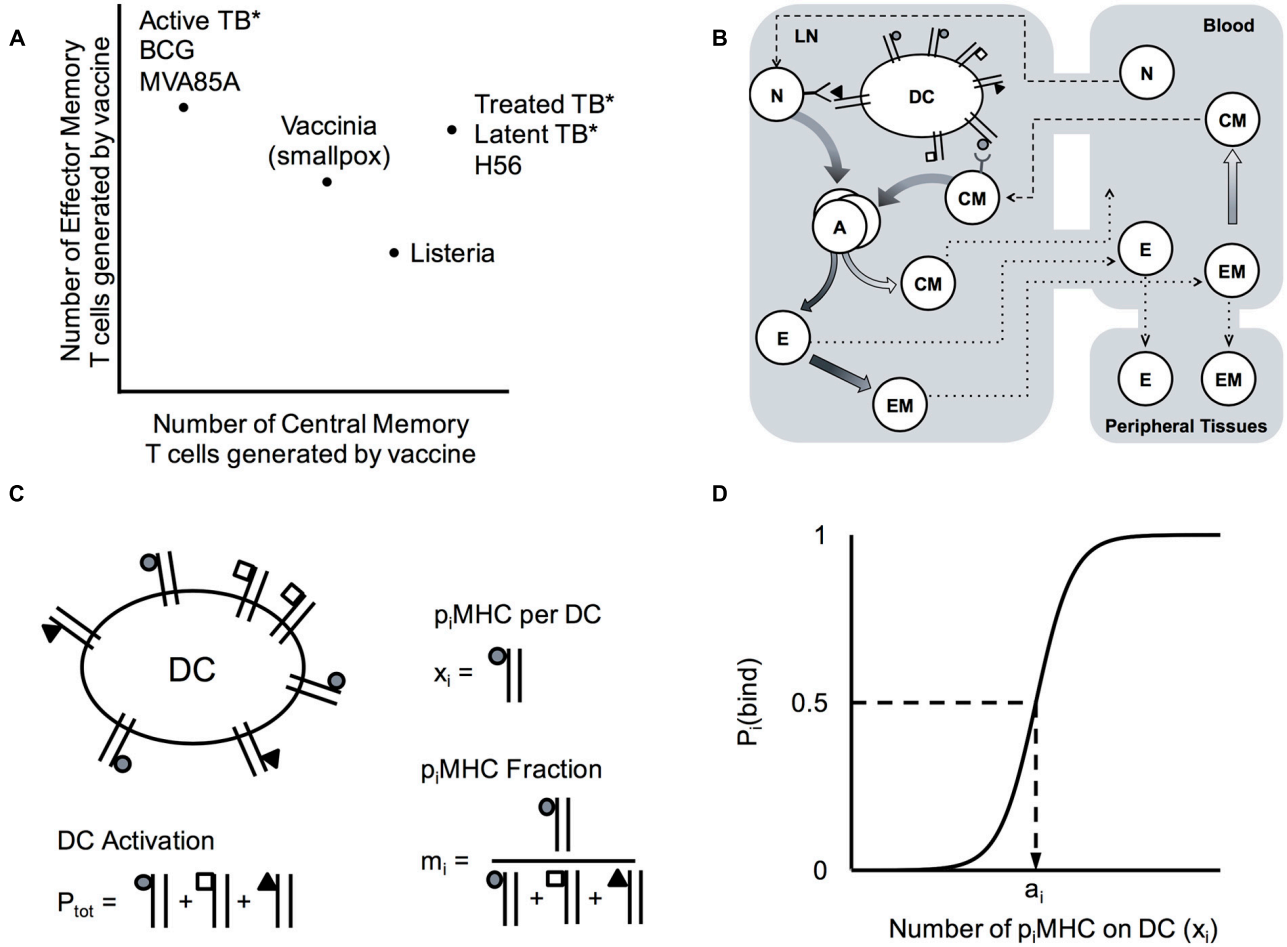
**Computational model system for predicting cell-mediated immune responses in Memory Design Space for multiple Ag specificities. (A)** Known cell-mediated immune responses generated by vaccine or natural infection to various infections. Reported relative numbers of central memory (CM) and effector memory (EM) T cells following vaccination are shown for smallpox in humans (6 months post-vaccination) (Miller et al., 2008) and listeria in mice (day 35) (Busch and Pamer, 1999; Pamer, 2004; Wong et al., 2004). Similarly, memory T cell populations generated following human vaccination with BCG (10 weeks) (Fletcher, 2007; Soares et al., 2008), MVA85A boosting BCG (24 weeks (Beveridge et al., 2007) or 56 days (Scriba et al., 2010)), and H56 (100 days after the 2nd of two boosts (Luabeya et al., 2015) are plotted, as well as T cells generated as a result of natural infection (marked by asterisks) with *M. tuberculosis* in patients with active TB disease (Goletti et al., 2006; Wang et al., 2010), latent TB infection (LTBI) (Wang et al., 2010), or successfully treated TB (1 month post-treatment) (Wang et al., 2010). We refer to a plot EM and CM T cells remaining in blood and peripheral tissues after infection has cleared and memory is established (time point *t =* 30 days) as *Memory Design Space*. Both the *size* and *skew* of the memory population can be easily visualized in Memory Design Space. **(B)** Schematic of computational model. Our 3-compartment hybrid model comprises an agent-based model of the lymph node and systems of ordinary differential equations representing blood and peripheral tissues. Ag-specific naïve T cells (N) in the LN may be primed by dendritic cells (DCs) and differentiate (graded arrows) to memory subtypes. In blood, cells may die, transit to other compartments, and some EM cells convert to CMs. Effector cells (E) and EMs that enter peripheral tissues do not reenter circulation. **(C)** Model quantities capturing antigen presentation. DCs display p_i_MHCs from several antigens (shown by different shapes). The number of pMHC complexes displayed on each Ag-bearing DC that enters the LN is P_tot_, the number of complexes for each antigen (p_i_MHC) is x_i_ and the fraction of total complexes for each antigen is m_i_. **(D)** Binding probability, P_i_(bind) describes the probability that a DC with an available pMHC receptor will bind a T cell in its neighborhood. This depends on x_i_, the number of p_i_MHC displayed that match the T cell’s specificity, and a_i_, the number of p_i_MHC necessary to achieve a 50% binding probability according to the equation: P_i_(bind) 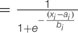, as was developed for previous versions of the model (Riggs et al., 2008; Linderman et al., 2010).

Due to the myriad ways pathogens have evolved to infect and propagate within their hosts, the optimal subtype composition of T cell memory populations varies across infections. We can conceptualize memory as a plot of EM vs. CM, for either CD4+ or CD8+ T cells, at a particular time point (**Figure 1A**). Protective vaccines against smallpox (Vaccinia virus) produce T cell populations that are comprised of slightly more EM than CM T cells (Miller et al., 2008), whereas protection against Listeria requires more CM (Busch and Pamer, 1999; Pamer, 2004; Wong et al., 2004). Natural infection with *M. tuberculosis* can lead to active disease, characterized by a memory cell population that is skewed toward EM in one study (Goletti et al., 2006; Wang et al., 2010). In the majority of cases, however, infection can be controlled (otherwise known as latent TB infection), and TB-specific memory populations are roughly balanced between EM and CM levels (Wang et al., 2010). Interestingly, T cells generated as a result of BCG vaccination have a very similar memory composition to active disease (Fletcher, 2007; Soares et al., 2008; Adekambi et al., 2012), but the new vaccine candidate H56 generates memory populations with approximately equal amounts of EM and CM T cells, similar to latent TB infection (Luabeya et al., 2015).

TB is a disease that typically lasts the lifetime of the host, and infection may lie on a spectrum from a truly latent infection to active disease. A host likely experiences several points on this spectrum over the timeframe of the disease, from early to later stages of infection. We theorize that an optimal memory cell subtype composition is different at each infection stage, as has been considered for other infections (Jiang et al., 2007). For example, T cells specific to antigens from the early phase of *M. tuberculosis* infection may be most effective as EM T cells, whereas Ag-specific populations associated with later stages may need to be skewed toward CM T cells to be effective. If so, an ideal vaccine would induce multiple Ag-specific populations of memory T cells, each with a distinct composition of CM and EM cells.

In order to achieve this, we consider immune mechanisms occurring within LNs that affect T cell priming in an Ag-specific manner. These mechanisms may be inherent to the specific interactions between T Cell Receptors (TCRs) and peptide-MHC complexes (pMHC) displayed on Dendritic Cell (DC) surfaces or they may be mechanisms that have different effects across Ag-specific populations. We are interested in general features intrinsic to a host’s immune response (e.g., length of time TCR binds pMHC (León et al., 2014) and vaccine delivery (e.g., dose and timing of stimulations), and specific features of the relevant antigens under study within a vaccine and how presented antigens interact with their cognate T cell (e.g., probability of TCR/pMHC binding).

Of the 100s of potential epitopes comprising a single invading pathogen, only a few tend to dominate the immune response that is mounted (Sant et al., 2005). Factors that determine this immunodominance hierarchy have been extensively investigated *in vivo* and *in vitro* for many pathogens, but remain poorly understood for TB. The frequency of naïve precursor T cells in a host is a strong predictor of response magnitude (Moon et al., 2007; Obar et al., 2008), but this is likely host-specific and difficult to affect with exogenous intervention. The probability of a binding event occurring between cells - a pMHC complex on the surface of an Ag-presenting cell and its cognate TCR on a naïve precursor T cell - is also strongly correlated with numbers of T cells generated (Busch and Pamer, 1998; Zehn et al., 2009; Bergsbaken and Bevan, 2015). Binding affinity and duration, for example, could potentially be altered, especially in the context of a subunit vaccine containing several antigenic peptides from the pathogen each of which can be manipulated independently.

The natural immunodominance hierarchy is convenient because it narrows the number of antigens in focus, but it may also be true that it is not the ideal hierarchy for fending off infection. Subdominant Ag-specific T cells could be more adept in controlling infection. In this case, we would desire a vaccine that could induce memory T cells that do not obey the natural hierarchy. Experimental evidence has shown that the natural immunodominance hierarchy can be disrupted by providing a second stimulation (boost) (Belz et al., 2000; Crowe et al., 2003; Jabbari and Harty, 2006; Kastenmuller et al., 2007; Handel and Antia, 2008) or by artificially forcing the system to over- or under- display certain antigens (Luciani et al., 2013; Woodworth et al., 2014). These perturbations have also been shown to affect the types of memory T cells that are generated following stimulation. For example, increasing the number of boosts can push the memory populations heavily toward EM T cell phenotype (Masopust et al., 2006; Wirth et al., 2010), while lowering the amount of total antigen presented (and thus the strength of signal) can cause the system to favor CM T cell generation (Kaech and Cui, 2012; Zehn et al., 2012; Gong et al., 2014).

In order to generate testable hypotheses regarding memory, and to account for combinatorial populations of T cells and interdependent mechanisms affecting their differentiation, we adopted a computational modeling approach. We updated our existing model of T cell priming and differentiation in LNs (*LymphSim*) to account for multiple Ag-specific populations of CD4+ and CD8+ T cells. This model also includes circulation of T cells between LN and blood (Gong et al., 2014). We extended this to include a compartment representing peripheral (non-lymphoid) tissues where T cells do not return to circulation once they leave (**Figure 1B**). We use this 3-compartment model to explore how both Ag-specific and Ag-independent mechanisms, some of which are specific to antigens and their cognate T cell populations and others of which apply to all cells in the LN equally, affect the numbers and types of memory T cells generated across several Ag-specific populations, focusing on CM and EM T cells. Recently a third T cell memory subtype has become recognized: resident memory T cells home to peripheral sites but do not reenter circulation (Mueller et al., 2013). It is thought that this subtype preferentially localizes to the initial infection site to act as sentinels (Masopust and Schenkel, 2013). Though distinct in their migratory patterns, some consider resident memory T cells to be a subset of EM T cells (Farber et al., 2014). In addition, specific markers and dynamic information about resident memory cells are currently lacking. Thus, in our studies we only focus on EM and CM subtypes, assuming that resident memory T cells are a subset of EMs.

As a simple way to distinguish Ag-specific immune cell populations with various compositions of CM and EM T cells, we conceived of *Memory Design Space*. As demonstrated in **Figure 1A**, each Ag-specific population is represented as a single point on an x-y plane, measured at a time after the acute immune response has cleared and only memory T cells remain, which we define as the *memory time point*. Numbers of CM T cells at the memory time point are plotted on the x-axis, and likewise the numbers of EM cells are plotted on the y-axis. Examining the plot of memory design space leads to two obvious measures: (1) The overall *size of the memory population* is represented by length of a line connecting the point to the origin; (2) The *skew of the memory population*, or the ratio of EM to CM cells, can be visualized by the angle created between this line and the x-axis. By plotting populations in Memory Design Space, we can easily visualize and compute the difference between several Ag-specific populations and thus assess how simulated conditions affect the amount and types of memory cells generated.

In this work, we use our computational model to explore how properties of LN environmental conditions, T cells, and antigens affect the amounts and types of memory T cells, i.e., location in Memory Design Space, generated from a virtual vaccine. We then ask how second or third rounds of vaccination (boosting) can influence these relationships. Given a target memory composition for each antigen and predictions generated by plotting model outputs in Memory Design Space, we believe it will be possible to harness relevant mechanisms to drive the outcomes in a desired direction. This can help improve efficacy of vaccines that induce cell-mediated immunity, especially those requiring protection by several Ag-specific populations like TB.

## Materials and Methods

### Interpreting Memory Populations Reported in Literature

Memory T cell population sizes reported in the literature were culled from multiple independent studies for specific pathogens, with priority given to studies in humans, non-human primates, and mice in that order (Busch and Pamer, 1999; Goletti et al., 2006; Miller et al., 2008; Petruccioli et al., 2013; Luabeya et al., 2015). All data were taken from FACS analysis of whole blood when available, or spleen. In some cases, the only counts of memory T cells available were from cytokine-producing cells, via intra-cellular cytokine staining. In these cases, we summed together the subpopulations (defined by combinations of cytokines produced) that authors identified as either EM or CM cells. Because of this and the disparate sources of data, we focused on the relative amounts of EM and CM T cells detected, rather than absolute counts.

### Hybrid Agent-Based Model of T Cell Priming and Differentiation

Agent-based models (ABMs) simulate stochastic processes evolving in both space and time. In our model, *LymphSim*, T cells and Dendritic Cells (DCs) are represented as discrete agents that move and interact on a three-dimensional grid representing a LN. The rules that govern cell behavior are defined over short distances and small time steps, and are probabilistic in nature. However, patterns of behavior can emerge on much larger scales of space and time. The cell interaction rules specified by this model lead to T cell priming and differentiation resulting from an infection or vaccine. Model rules are available in the supplement and are briefly summarized below.

In this work we built upon our previously developed model of T cell priming and differentiation in LN and circulation via blood (Gong et al., 2014). The LN compartment is represented with a 3-dimensional hybrid ABM, called *LymphSim*. The grid spaces (cubes of side length 20 μm) are arranged in a truncated cone, which represents approximately 1/200th of a LN. T cells and Dendritic Cells (DCs) are the model agents: DCs move randomly on the grid and T cells move in a persistent random walk at rates calibrated to intravital 2-photon microscopy experiments (Miller et al., 2004; Gong et al., 2013). T cells and DCs enter and exit the grid at locations designated as high endothelial venules and efferent lymphatics, respectively. These ports connect the LN to the blood compartment (**Figure 1B**), which is represented by ordinary differential equations (ODEs) that are described in a following section. The LN and blood compartments share information and update at the end of every ABM time step (25 simulated seconds), an important step because cells that will enter the LN are recruited at rates dependent on their blood concentrations, and cells that have exited the LN contribute to blood concentrations.

The rules for *LymphSim* have been published and are available at http://malthus.micro.med.umich.edu/lab/movies. Briefly, T cell – DC binding is permitted to occur when the two cells are in neighboring grid compartments and there is open space on surface of the DC to accommodate the T cell. Once bound, a T cell accumulates stimulation from the DC at a rate proportional to the total number of pMHC displayed on that DC (Gett and Hodgkin, 2000; Gong et al., 2014; Moreau and Bousso, 2014). The amount of accumulated signal is used to determine whether the T cell will ultimately differentiate into an EM or CM T cell, or whether it will return to its naïve resting state.

Accumulated signal is the main factor determining T cell fate after unbinding from a DC. Mechanisms that determine accumulated signal for each DC-T cell interaction may either be independent of the antigen (Ag) involved, affecting all interactions equally in the LN, which we term *Ag-independent*, or what we term *Ag-specific*, i.e., uniquely specified for each population of cognate T cells and their interactions. DCs in the model are promiscuous, displaying more than one antigen at a time as they do in nature. The total number of pMHC displayed per DC, P_tot_, is an *Ag-independent property* because of this lack of Ag-specificity and because all Ag-bearing DCs (AgDCs) entering the LN have the same initial P_tot_. This quantity serves as a proxy for the overall activation state of a DC, as DCs displaying higher numbers of total pMHC (having a higher P_tot_) pass more stimulation signals to their bound T cells. *Ag-specific properties* include the binding kinetics of a T cell Receptor (TCR) to the peptide-MHC complex displaying its cognate antigen *i* (p_i_MHC), which are discussed in detail below. Additionally, the presence and quantity of an antigen being presented by DCs can be controlled independently for each antigen, simulating varying dose and number of rounds of vaccination.

An important distinction between differentiated T cell subsets is their migration patterns: only Naïve and CM T cells may enter a LN, whereas after emerging from the LN, Effector and EM T cells circulate through blood and may enter peripheral tissues, from which they do not return (Mueller et al., 2013). Additionally, CM activation upon antigen re-encounter happens more readily than Naïve cell activation, and as reported in the literature, CM cells accumulate signal from DCs more efficiently (Byrne et al., 1988; Bachmann et al., 1999).

### Multiple Antigen-Specific T Cell Populations in the LN

For this work, we expanded our model of T cell priming and differentiation (*LymphSim)* to account for multiple Ag-specific T cell populations as described in the Model Rules (Supplementary Data S1). Briefly, The TCR of each T cell agent recognizes only its cognate p_i_MHC complex. For the simulations shown here, we used five Ag-specific T cell populations (*i = 1,2,…,5*) for each of CD4+ and CD8+ T cells, a total of ten Ag-specific populations. We assume that the vast majority of dynamics within the LN are similar across all Ag-specific T cell populations, but, as has been reported in the literature, certain properties differ between Ag-specific populations. These include precursor frequency (Moon et al., 2007; Obar et al., 2008), number of p_i_MHC displayed on a DC (Borghans et al., 1999; Gonzalez et al., 2005; Baumgartner et al., 2010; Huang et al., 2013), and TCR-p_i_MHC binding probability (Kotturi et al., 2008; Chervin et al., 2009; Irving et al., 2012). In our model, precursor frequency is a single parameter specified independently for each population of Ag-specific CD4+ and CD8+ Naïve T cells. The number of p_i_MHC displayed per DC is specified as a fraction of total pMHC-I or pMHC-II displayed per DC (*m_i_*) (**Figure 1C**).

When a T cell comes within the binding radius of a DC, the probability of a binding event occurring, P_i_(bind), is determined by the number of cognate p_i_MHC displayed on the DC. The binding probability equation was developed previously (Riggs et al., 2008; Linderman et al., 2010) and here is updated to represent Ag-specific binding reactions when more than one Ag-specific population of T cells is present:

$$P_{i}(bind)=\frac{1}{1+e^{-\frac{\left(x_{i}-a_{i}\right)}{b_{i}}}}$$

where *x_i_* represents the number of p_i_MHC displayed on the DC, *a_i_* represents binding threshold, and *b_i_* is binding slope (**Figure 1D**). While binding probability P_i_(bind) depends only on the cognate p_i_MHCs, stimulation signal accumulated by bound T cells depends on the level of DC activation, which is approximated in our model by the total pMHC (P_tot_) displayed regardless of Ag-specificity.

Each T cell-DC binding event in *LymphSim* represents the sum of several serial bindings between a T cell and several DCs in a real LN. Data suggest that the binding time for each event in the series is a function of several Ag-independent mechanisms including crowding, DC activation state, T cell stimulation experience, and other conditions in the LN combined with Ag-specific properties: a T cell’s binding affinity to its cognate p_i_MHC and the number of cognate p_i_MHC displayed (Bousso and Robey, 2003; Celli et al., 2005, 2007, 2008, 2012; Garcia et al., 2007; Henrickson et al., 2008; Moreau and Bousso, 2014). We use binding time to refer to the Ag-independent conditions that affect the total time a T cell collects stimulation from DCs while it is in the LN. We do not explicitly model Ag-specific DC-T cell binding affinity, but Ag-specific binding probability influences overall stimulation collected by modulating whether or not an Ag-specific T cell will bind its cognate p_i_MHC when in proximity of a DC.

After exiting the LN, CMs (that may later reenter and bind another DC) retain the accumulated stimulation signal they received. This so-called “stimulation history” is one mechanism by which CMs are more easily primed than their naive counterparts (Byrne et al., 1988; Bachmann et al., 1999). Because stimulation history depends on Ag-specific interactions, the amount of stimulation signal accumulated is another Ag-specific property. While in the LN, each CM cell tracks its own amount of accumulated stimulation. In the blood, where cells are not tracked individually but as a population (see next section), the mean and standard deviation of stimulation on CMs in each Ag-specific population is updated each time a new CM exits the LN. CMs returning to the LN are assigned a value of accumulated signal that is sampled from the normal distribution defined by their population’s mean and standard deviation.

### Multiple Antigen-Specific T Cell Populations in Peripheral Tissue and Blood

The blood compartment is represented by ODEs that track concentrations (cells/μL) of subtypes of CD4+ and CD8+ T cells (naïve, effector, CM, EM). The equations include terms that account for homeostatic proliferation and/or death rates, egress to peripheral tissues, and a source of new naïve cells generated in the thymus. To track Ag-specific populations, we generated a new instantiation of the base blood ODE model for each Ag building on our previous published version (Gong et al., 2014). The equations and parameter values are the same for each, but each population is updated independently as cells exit and enter the blood compartment. Essentially, the blood compartment model is comprised of six parallel, independent sets of ODEs: one for each of five Ag-specific population of T cells and one for all other (non-cognate) T cells.

We added an additional ODE compartment representing peripheral non-lymphoid tissues (“NLT” in the equations below; **Figure 1B**). This compartment represents tissues where infections are likely to occur, such as lungs, but keeps the model formulation general enough that we can consider a generalized infection scenario and still explore the role of trafficking patterns between compartments. Only effector and EM T cells may enter peripheral tissues, and no cells exit. Cell population dynamics are tracked as an exponential decline of both effector and EM cells. Similar to the blood compartment, both CD4+ and CD8+ T cells of each TCR type are described by their own set of equations so that the dynamics are tracked separately for each Ag-specific population. The equations representing dynamics of one Ag-specific population (Ag1-specific T cells) for CD4+ and CD8+ T cells are shown here:

$$\begin{array}{c} \frac{dE_{4,1,NLT}}{dt}=\xi_{E4,1}E_{4,1,B}-\delta_{E4,1,NLT}E_{4,NLT}\;\\ \frac{dEM_{4,1,NLT}}{dt}=\xi_{EM4,1}EM_{4,1,B}-\delta_{EM4,1,NLT}EM_{4,1,NLT}\\ \frac{dE_{8,1,NLT}}{dt}=\xi_{E8,1}E_{8,1,B}-\delta_{E8,1,NLT}E_{8,1,NLT}\;\\ \frac{dEM_{8,1,NLT}}{dt}=\xi_{EM8,1}EM_{8,1,B}-\delta_{EM8,1,NLT}EM_{8,1,NLT} \end{array}$$

where ξ_E4,1_ and ξ_EM4,1_ are rates of Ag 1-specific CD4+ effector (E_4,1_) and EM (EM_4,1_) being recruited from blood to peripheral tissues; δ_E4,1,NLT_ and δ_EM4,1,NLT_ are the death rates of Ag 1-specific CD4+ effector and EM in the peripheral tissues and the terms for Ag 1-specific CD8+ T cells follow the same conventions. The linked LN, blood, and peripheral tissue models formed by ABM and ODE models, together constitute a hybrid ABM that is used for simulations of T cell priming and differentiation.

### Parameter Estimation

**FIGURE 2:**
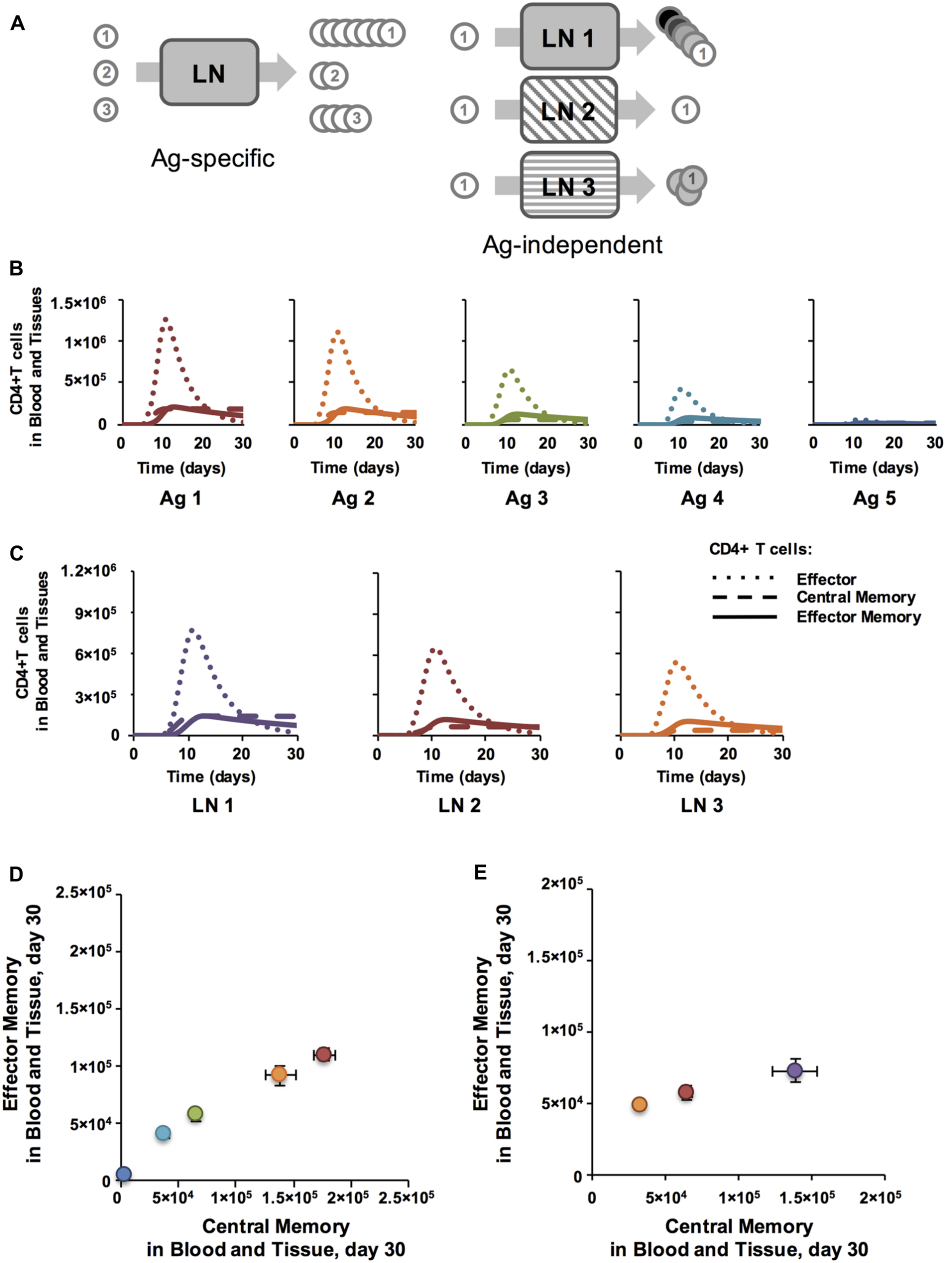
**Antigen specific characteristics affect Memory Population Size and antigen independent mechanisms affect EM/CM ratio for CD4+ T cells. (A)** Simulation set-up: Ag-specific mechanisms have varying affects on Ag-specific T cell populations (numbered circles) within a single LN. Ag-independent mechanisms, here conceptualized as three different LN environments, cause variations in cells from the same Ag-specific population. **(B)** Binding probability affects the number of CD4+ T cells generated in LN. *y*-axis: total numbers of T cells in blood and peripheral tissue. *x*-axis: days post-vaccination. CD4+ T cell populations were assigned increasing binding thresholds (a_i_) from low (Ag1-specific T cells) to high (Ag5-specific T cells) such that the binding probability, P_i_(bind), of Ag1-specific T cells was highest, followed by Ag2-specific T cells, and so on with Ag5-specific T cells having a very low chance of binding their matching p_i_MHC presented on the surface of a DC. All parameters were at baseline values, except binding thresholds, which were set at 30, 90, 150, 200, 300 for Ag1-Ag5, respectively. **(C)** Binding time affects amount of CM, but not EM, generated. Numbers of CD4+ T cells in blood and tissue are plotted post-vaccination for 3 simulations, with DC-T cell binding time (*max binding time Naïve*) increasing from LN 1 (4 h) to LN 2 (8 h) to LN 3 (12 h). **(D)** Memory T cell populations at 30 days post-vaccination corresponding to time courses in panel B are plotted in Memory Design Space. Colors show binding probability from low (blue) to high (red) and correspond to graphs in Panel B. Error bars represent SEM (*n* = 10). **(E)** T cell populations measured 30 days post-vaccination in blood and peripheral tissues corresponding to time courses in panel C are plotted in Memory Design Space. Purple: LN 1, Red: LN 2, Orange: LN 3. Error bars represent SEM (*n* = 10). See Supplementary Figure S1 for statistical analysis of change in skew.

Because most processes described by model parameters were unchanged by the addition of multiple antigen specificities and a peripheral tissue compartment, most baseline parameter values were not varied from the values in the single antigen version of the model (Gong et al., 2014). However, we confirmed that the numbers and dynamics of T cells exiting the LN were also unchanged. We compared the qualitative dynamics of T cells in the blood following antigen presentation (e.g., **Figures 2B,C**) to data from mouse spleens presented in (De Boer et al., 2001, 2006; Antia et al., 2003; Ganusov and De Boer, 2007). We expected to see a large peak of effector T cells that reached its maximal value 7-10 days post-infection and quickly declined and memory T cells that reach lower peaks but declined more slowly, especially for CM. We also used the mouse data as a lower bound for the numbers of T cells generated. To facilitate comparison to data, T cells from the model are reported as absolute numbers: total cells in the blood and peripheral tissues. In addition, we verified that the size of the memory populations generated by the model were approximately 5-10% of the peak of the effector response generated, as has been reported widely in the literature (De Boer et al., 2001; Badovinac et al., 2003). The simultaneous priming of multiple antigen specificities did not interfere with T cell dynamics or population sizes generated per antigen for the parameter ranges tested here.

Baseline values of Ag-specific parameters such as precursor frequencies and binding parameters were previously estimated for a single antigen, and many remained unchanged (Gong et al., 2014), but are now specified for five distinct populations. For the five Ag-specific populations in our model, we set each *m_i_* ≤ 0.2 so that their sum is less than 1; presumably other pMHC complexes not relevant to the infection of interest also are present and would constitute the balance. All baseline parameter values for Ag-specific and Ag-independent parameters are listed in Supplementary Table S1. If values are used for parameters other than those listed, we give them in the Figure legends.

### Uncertainty and Sensitivity Analyses

We used uncertainty and sensitivity analysis to identify correlations between model parameters and memory population size and/or EM/CM ratio. To do this, we used the skew in Memory Design Space to indicate the ratio of EM to CM, and distance in Memory Design Space to indicate size of the memory population. Latin Hypercube Sampling (LHS) was used to efficiently sample parameter space, generating 50 parameter files that were each simulated 20 times with a unique random seed. Correlations between parameter values and skew/distance were estimated with Partial Rank Correlation Coefficients (PRCC; Marino et al., 2008). To determine statistical significance of the PRCC correlations, we performed a pairwise *Z*-test between all PRCCs.

### Multiple Vaccination Events

We simulated two types of vaccination events: prime, where the vaccine is introduced into a naïve host with no previous antigen exposure, and boost, where the system has previously encountered the same antigens that are being presented. To simulate priming, the model is run for 2 days to allow initialized populations to reach steady state, and antigen presentation begins on day 3. Vaccination is captured in our computational model by the entry of AgDCs into the LN. AgDCs are recruited steadily for two days as space permits. DCs do not exit LNs, but their lifespan is 3-5 days (Kamath et al., 2002), and therefore the antigen presentation period naturally resolves within 7 days for a 2-day recruitment period.

We simulated boosts in two ways: either by extending priming simulations for several weeks beyond the initial memory time point and reintroducing antigen for an additional identical round of antigen presentation, or by setting initial conditions to represent a boost scenario, with Ag-specific memory populations already present in blood and LN before a simulation begins. To do this we had to endow CM T cell populations with stimulation signal, indicating the average stimulation history for each Ag-specific population (see above). This information is represented by a mean and standard deviation for each Ag-specific population of CMs in the blood.

### Generating Desired Responses for TB Infection Studies

To demonstrate that diverse T cell populations could be generated with our computational model, using a reverse engineering approach we identified “desired responses” of varying size and EM/CM ratio and then performed simulations designed to generate T cell populations that met our specifications. Our choices of desired responses were guided by EM/CM ratios and T cell numbers reported in literature (Goletti et al., 2006; Fletcher, 2007; Soares et al., 2008; Wang et al., 2010; Adekambi et al., 2012) (Luabeya et al., 2015; **Figure 1A**). From our previous analyses we knew that low binding time led to high numbers of CM relative to EM (**Figure 2C**) and likewise, high CM efficiency generated the opposite (**Table 1**). Furthermore, as demonstrated in **Figure 2D**, binding threshold is anti-correlated with population size, and number of boosts is correlated with both size and EM/CM ratio. Therefore, we reasoned, there should exist some combination of those parameters that allows us to reach almost any region in Memory Design Space. We varied parameters simultaneously using LHS and selected parameter sets that generated memory populations most closely matching the previously defined desirable responses. To determine the best matching simulations, we required that the skew be within 22.5° (π/4 radians) of the desired response and the population size be within a window of ±20%.

**Table 1:** Sensitivity and uncertainty analysis for skew and population size.

	Ag 1	Ag 2	Ag 3	Ag 4	Ag 5
**CD4+ T CELL EM/CM RATIO**					
CD4+ prob Effector Memory (EM)	+++	+++	++	++	++
CD4+ median effector prob	-	+	-	-	-
CD4+ efficiency Central Memory (CM)	+++	+++	+++	+	
CD4+ cm binding Time	++	+	+		
Initial pMHC MDC (P_tot_)	++	++			
CD4+ median priming prob	++		+	+	
CD4+ max binding time Naive				++	++
**CD8+ T CELL EM/CM RATIO**					
CD8+ max binding time Naive	+++	+++	+++	+++	++
CD8+ median effector prob	---	---	---	--	-
CD8+ median priming prob	++	++	++	++	+
CD4+ max binding time Naive	-	-	-	-	
CD4+ median priming prob	-		-	-	-
Initial pMHC MDC (P_tot_)	++	+			
**CD4+ MEMORY POPULATION SIZE**					
CD4+ median priming prob	---	--	--	--	-
CD4+ median effector prob	+++	+++	+++	++	++
CD4+ max binding time Naive	--	--	---	---	-
Max Number DCs	++	++	++	++	++
Initial pMHC MDC (P_tot_)	---	--		++	+++
CD4+ efficiency CM	---	---	--		
CD4+ cm binding time	--	-			
**CD8+ MEMORY POPULATION SIZE**					
Max number DCs	++	++	++	++	+++
CD8+ median priming prob	--	--	--	-	-
CD8+ max binding time Naive	---	---	--	-	
CD8+ median effector prob	++	++	++	+	
Initial pMHC MDC (P_tot_)	-			++	+++
***p*-values:**					
0.05 > *p* > 0.001	+/-				
0.001 > *p* > 10e-6	++/--				
10e-6 > *p* > 0	+++/---				

*As described in section “Materials and Methods,” uncertainty and sensitivity analysis was performed by calculating Partial Rank Correlation Coefficients over simulations with parameter values sampled by Latin Hypercube Sampling. Pluses and minuses indicate *p*-value ranges as indicated; parameters with *p*-value < 0.05 are included for each outcome. Parameter definitions and ranges of all parameters varied are found in Supplementary Table S1. Binding thresholds (a_i_) for each Ag-specificity are: 30, 90, 150, 200, 300 for Ag1-Ag5, respectively.*

### Model of Granuloma Formation and Function in TB

The immune response to *M. tuberculosis* infection typically results in the formation of cellular structures termed granulomas in the lung. Granulomas serve to physically contain and immunologically restrain bacteria over months to years of infection. If granulomas cannot contain the infection, active TB results. We use our well-established computational model of granuloma formation and function, *GranSim*, to capture the dynamics of infection with *M. tuberculosis* in the lung (Segovia-Juarez et al., 2004; Fallahi-Sichani et al., 2011; Cilfone et al., 2013, 2015; Pienaar et al., 2015). Briefly, the model, a hybrid ABM, describes cellular behavior, including recruitment to the lung, changes of state (activation, infection, etc.), and movement. Cells (agents) are macrophages and T cells (CD4+, CD8+, and regulatory T cells) that can have multiple states and phenotypes (e.g., infected, activated). Three populations of bacteria (intracellular replicating, extracellular replicating and extracellular non-replicating bacteria) are represented as continuous functions in the extra- or intra-cellular environment. Probabilistic interactions between immune cells and bacterial populations are described by a well-defined set of rules between immune cells and *M. tuberculosis* in the lung that are continuously updated based on new biological data. We describe the diffusion of relevant chemokines, cytokines, and other soluble ligands (e.g., anti-TNF antibodies) by solving the relevant partial differential equations. All rules for *GranSim* are available at http://malthus.micro.med.umich.edu/lab/movies. Each granuloma simulation follows events over several hundred days, building over time to track 1000s of individual cells. In the simulations used here, we capture a 600 μm^2^ cross-section of lung tissue.

### T Cell Immunity for Preventing TB Infection and Enhancing Granuloma Clearance

#### Model System

To see the impact of a pool of memory T cells during a long time course of *M. tuberculosis* infection, we used *GranSim*, our computational model that captures dynamics of TB granuloma formation in lungs, and interfaced it with two other physiological compartments represented by ODEs describing blood and LNs (Linderman et al., 2015). Briefly, in this model implementation, T cells are recruited to the lung in proportion to their concentrations in blood. These concentrations are determined by LN output following antigen presentation.

#### Vaccination

Memory T cells may be present in blood because of a previous infection or a vaccine. We simulated vaccination with TB-specific antigens by specifying non-zero initial concentrations of TB-specific memory T cell in the blood ODEs of *GranSim*. Next, Mtb infection was simulated in the lung, and granuloma formation was followed for 300 days to test whether there was increased protection when compared to simulated natural infection (without vaccination). H56 is a multi-subunit TB trial vaccine containing antigens specific to both early and late stage infection (Aagaard et al., 2011; Lin et al., 2012). We implemented this concept in our simulations by introducing a second (late-associated) Ag-specific population of T cells; i.e., at a chosen time point we allow a specified number of non-cognate memory T cells to convert to cognate memory cells and then continue to allow granuloma formation and function to evolve.

Outcomes were defined with respect to number of *M. tuberculosis* according to the following criteria: (1) If the number of bacteria dropped below 1 by day 10, the granuloma is categorized as never having formed, “No Infection Established;” (2) If the number of bacteria in the granuloma dropped below 1 by day 300, the granuloma is categorized as “Cleared”; (3) if the number of bacteria at day 300 was greater than 2000, the granuloma was categorized as “Disseminating infection”; (4) otherwise, the granuloma was categorized as “Contained.” If a granuloma was contained (stable) and was perturbed to dissemination, we say that granuloma reactivated. Outcomes were examined with and without vaccine, and with and without anti-tumor necrosis factor (anti-TNF). Anti-TNF reduces the inflammatory response and is known to cause reactivation TB in both experiments and our simulations (Lin et al., 2010, 2012). Note that the outcome measures are not at the host scale, but at the granuloma scale.

We wanted to assess protective qualities of memory T cells against (a) establishing infection and (b) reactivating latent infection. In all cases, *M. tuberculosis* was given at day 0. Two control groups were used: Group 0 received no vaccine and no anti-TNF while Group T0 received no vaccine, but was treated with anti-TNF 200 days post-infection. To estimate the benefit of vaccination, two experimental groups were compared to each control. Groups 1 and T1 received memory T cells prior to infection on day 0. These CM and EM CD4+ and CD8+ T cells represent a vaccine with antigen specific to early stages of *M. tuberculosis* infection. Groups 2 and T2 received the early specific memory cells at day 0, and also received an additional set of memory T cells on day 150. This simulated a second memory T cell population specific to later phases of infection and also included both CD4+ and CD8+ CMs and EMs. Groups T1 and T2 received anti-TNF on day 200 to test the protective effects of T cells against reactivation, while groups 1 and 2 did not so as to be comparable to Group 0.

We hypothesized that size and skew of memory populations would affect infection control and reactivation. Accordingly, we used uncertainty analysis by applying an LHS to vary numbers of CD4+ and CD8+ CM and EM T cells initialized in the blood at days 0 and 150, generating 50 unique vaccine formulations, each consisting of four Ag-specific memory populations (CD4+ Early, CD8+ Early, CD4+ Late, CD8+ Late) with a unique EM/CM ratio and size. We defined a baseline (natural infection) parameter set to be one that consistently led to containment without further intervention (Group 0) and consistently reactivated upon TNF inhibition (Group T0). Using the same baseline parameter file, we simulated each of the 50 vaccine formulations by initializing early specific T cells in the blood at day 0 (Groups 1, 2, T1, and T2) and for Groups 2 and T2, initializing additional late-specific T cells at day 150. We compared outcomes to Groups 0 or T0: in Groups 1 and 2 we determined formulations that led to high levels of clearance, and in Groups T1 and T2 formulations that increased containment over the respective controls. The five formulations with the most improved outcomes were simulated 100 times per group and the most successful formulation is shown in the results.

### Model Implementation

Our hybrid models are implemented in C++ with Boost libraries (distributed under the Boost Software License – available at www.boost.org). Each model can be run with or without graphical user interface (GUI) visualization and can be run on Linux, Mac OS, and Windows platforms. The GUI for *GranSim* was built using the Qt framework (open-source, distributed under GPL – available at qt.digia.com), which allows us to display, track and plot different readouts of the *in silico* simulation in real-time. For *LymphSim*, a forward Euler method is used to solve the ODEs. Each time step of the LN ABM simulation is divided into 100 pieces (step size of 0.25 s) to reduce error. On the XSEDE Stampede system at University of Texas, each *LymphSim* simulation of 30 days (LN sub-model is active for ∼20 days) takes 12-20 h to run and each *GranSim* simulation (including LN and Blood ODEs) of 300 days takes ∼30-45 min to run.

## Results

In these studies, we captured T cell priming and differentiation dynamics and the status of memory cell levels following vaccination. We performed two types of studies (**Figure 2A**). In the first, we simulated generation of five Ag-specific populations of T cells simultaneously in a LN (**Figure 2A**, left), varying only Ag-specific parameters in the LN (Ag-specific versus Ag-independent parameters are identified in Supplementary Table S1). We asked which properties intrinsic to those antigens are important in determining amounts and types of memory T cells generated, i.e., location in Memory Design Space. In the second, we varied Ag-independent characteristics, e.g., DC Activation (P_tot_), DC - T cell binding time, and priming threshold, to name a few, in simulations with five Ag-specific populations, to ask which Ag-independent characteristics of a LN affect location in Memory Design Space. These studies allowed us to apply the model to address questions related to TB vaccine development. We then performed analysis to determine which features of the model control differences in memory cell generation and applied our knowledge to explore TB vaccination strategies.

### T Cell-pMHC Binding Probability Correlates with Size of Memory Populations Generated

We first asked how Ag-specific parameters determine EM/CM ratio (skew) and overall size of Ag-specific memory populations (**Figure 2A**). We varied the binding probability between an Ag-specific T cells and a DC presenting cognate antigen by assigning different binding threshold (*a_i_*) values to 5 Ag-specific populations. All other parameters were held constant at baseline values, whether they were Ag-specific or Antigen independent. **Figure 2B** shows time courses of all Ag-specific CD4+ T cells in the blood (including peripheral tissues); CD8+ dynamics are not shown, but similar. The dynamics of each cell type in the blood are similar across Ag-specific populations: effector T cells enter by day 5, peak by day 10, and soon die off, leaving behind EM and CM CD4+ T cells. However, the size of the response decreased with decreasing binding probability (increasing binding threshold, **Figure 2B**). To assess the characteristics of the memory populations generated, we measured the number of each memory CD4+ T cell subtype remaining in blood and peripheral tissues at 30 days post-vaccination, our memory time point, long after the immune response has waned and plot these in Memory Design Space (**Figure 2D**). The size of the memory populations decreased with decreasing binding probability, as expected. Interestingly, the skew in Memory Design Space was not affected by varying binding probability: a relatively constant ratio of EM to CM T cells is generated across all Ag-specific populations despite dramatically different total numbers of Ag-specific memory CD4+ T cells.

### DC-T Cell Binding Dynamics Correlate with Ratio of EM to CM Cells Generated

To examine how Ag-independent mechanisms affect EM/CM ratio and memory population size, we compared how a single Ag-specific population of T cells fares across multiple LN environments with varying Ag-independent characteristics (**Figure 2A**). As an example of an Ag-independent mechanism, we varied DC-T cell binding time (*max binding time Naïve* in Supplementary Table S1), a parameter that represents the dwell time of a T cell across what might be several DC binding events (see Materials and Methods). While both Ag-specific and Ag-independent properties have been attributed to binding time in the literature (Moreau and Bousso, 2014), here we capture the Ag-specific portion of binding time with binding probability, and binding time refers to the Ag-independent effects that affect the total time a T cell collects stimulation from DCs while it is in the LN. Time courses of CD4+ T cell effector, CM, and EM generation across three different LN that have differing binding environments (i.e. different binding times) are shown in **Figure 2C**; the patterns hold across Ag-specific populations with different binding probabilities and also in CD8+ populations. Interestingly, while the number of CM T cells generated decreases with binding time, the number of EM T cells generated is not correlated with binding time (**Figure 2E**). This is due to a process described in section “Materials and Methods”: T cells bound to DCs accumulate stimulation, which decreases the likelihood that they will differentiate into a CM T cell as opposed to an Effector T cell, some which may later become EM T cells. This trend remains true at the memory time point (day 30), so we see that both the skew and size of the memory population are affected (**Figure 2E**). This means that LNs whose Ag-independent factors induce longer T cell-DC binding times can generate memory T cell populations with a greater proportion of EM T cells when compared to Ag-independent factors that lead to short T cell-DC binding times. Thus, we have identified an Ag-specific mechanism that primarily affects the size of the memory population (binding probability) and an Ag-independent mechanism that affects both the skew and size (binding time). Is it possible to simultaneously affect both across Ag-specific populations? If so, multiple Ag-specific memory populations with diverse skew and size could be generated from the same vaccination.

### LN Environmental Conditions Influence EM/CM Ratio and Size of Ag-Specific Memory Populations

To determine which Ag-independent mechanisms significantly influence memory population size, skew in Memory Design Space, or both, we performed a sensitivity and uncertainty analysis. **Table 1** lists the parameters most significantly correlated with size and EM/CM ratio (skew) for five Ag-specific populations of both CD4+ and CD8+ memory T cells. Interestingly, binding time is correlated with skew for CD8+ but not CD4+ T cells. In our model, we assume that CD8+ T cells are only able to be primed by Licensed Dendritic Cells (Wiesel and Oxenius, 2012), which means that they are able to receive more stimulation per time step once bound. This increase in efficiency contributes to the difference in significance between these two cell types. Indeed, other parameters governing priming efficiency (e.g., CM efficiency and DC activation level) are significantly correlated with skew in Memory Design Space for CD4+ T cells. Effector Threshold (*median Effector Prob*), which governs primed cell differentiation into either Effector (including EM precursors) or CM, is negatively correlated with skew in Memory Design Space for both CD4+ and CD8+ T cells. A higher threshold indicates a higher probability of CM cells being produced, which translates to a smaller skew in Memory Design Space. This parameter is also significantly correlated with size in Memory Design Space. As the threshold increases, more CMs are produced, which increases the pool of cells that can be primed. In general, each mechanism correlates with either EM/CM ratio or memory population size, but not both.

### Identifying a Broad Range of EM to CM T Cell Ratios Across Ag-Specific T Cell Populations with Different Binding Probabilities

**FIGURE 3:**
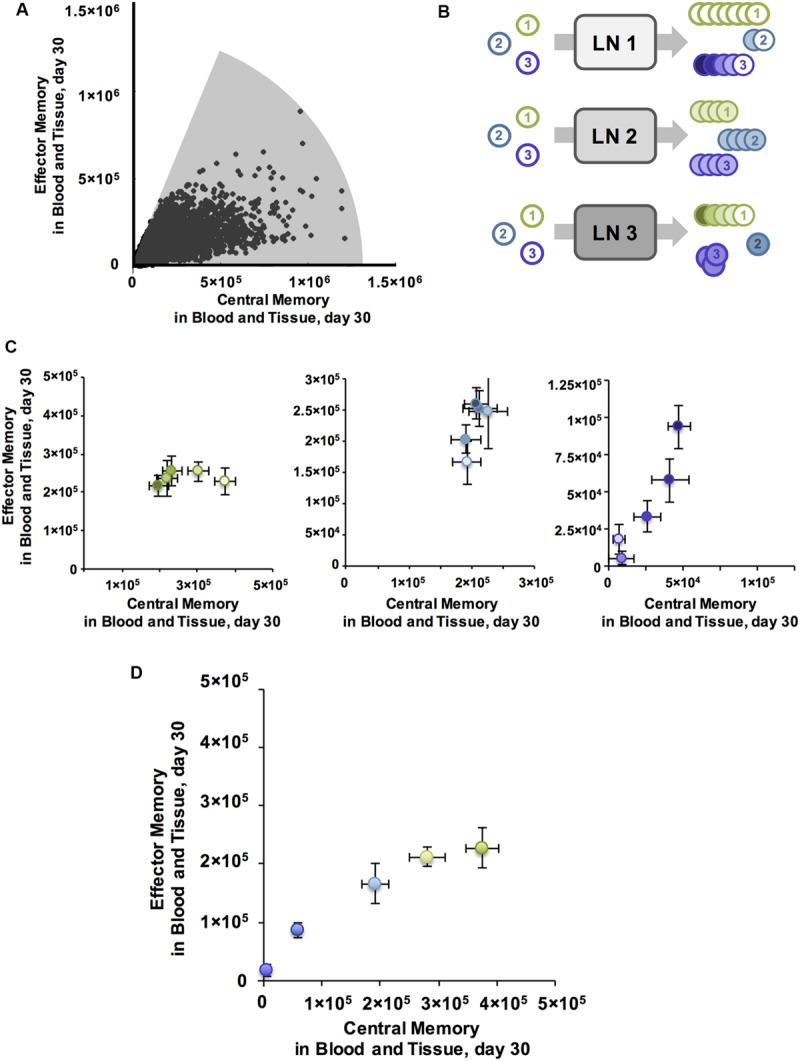
**Antigen independent mechanisms can have varying affects across antigen specific CD8+ T cell populations, producing memory populations with a range of characteristics. (A)** A single LN, wherein all Ag-independent parameters are constant, can generate a variety of EM/CM ratios and memory population sizes across Ag-specific T cell populations. Shaded area represents region of CD8+ Memory Design Space reached after simulating parameter sets sampled by sensitivity analysis. **(B)** Ag-independent mechanisms may have differing affects across Ag-specific populations. We can compare differences in Ag-specific responses across LNs or Ag-independent responses from distinct LNs across Ag-specific populations. **(C)** Ag-specific binding probability can influence the direction of correlation with P_tot_. For CD8+ T cell populations with high binding probability P_tot_ is anti-correlated with EM/CM ratio. Leftmost panel plots Ag-specific memory populations in Memory Design Space. Each T cell population simulated had high P_i_(bind) with binding threshold a_i_ = 30, and was generated from one of five LNs, with increasing P_tot_ (light to dark). All error bars represent SEM (*n* = 10). For T cells with medium binding probability (a_i_ = 150, middle panel, blue), P_tot_ is positively correlated with both EM/CM ratio and size of memory population. As on left, each population was generated from a separate LN with increasing P_tot_, but Ag-specific populations with medium P_i_(bind) are shown here. For T cells with low binding probability (a_i_ = 300, right panel, purple), P_tot_ is positively correlated with EM/CM ratio and size of memory population. For all panels, P_tot_ values were 100, 200, 300, 400, 500. **(D)** With all Ag-independent parameters held constant, the parameter set that generated the greatest diversity of EM/CM ratios across Ag-specific populations is plotted in Memory Design Space. P_i_(bind) varied from high to low across T cell populations (purple = low, green = high). Error Bars represent SEM (*n* = 10).

We hypothesize that it would be advantageous to harness mechanisms that allow generation of a wide range of EM/CM ratios and memory population sizes, i.e., covering as much of Memory Design Space as possible, to fight various infections. We plotted every memory T cell population that resulted from the thousand simulations used for our sensitivity and uncertainty analysis (**Table 1**) in a single Memory Design Space. **Figure 3A** shows the regions of Memory Design Space reachable by CD8+ populations when varying Ag-independent and Ag-specific mechanisms together. CD4+ populations covered a similarly shaped region, but fewer round of Effector proliferation led to less coverage of the y-axis for these populations (not shown). Even with CD8+ populations, we were unable reach the upper left corner of Memory Design Space, especially when compared with **Figure 1A**. Even so, we wanted to know the how much of Memory Design Space could simultaneously be reached with different antigens (e.g., present within a single vaccine). So, we asked which Ag-independent parameters could have unequal effects across Ag-specific populations with varying binding probability (**Figure 3B**). From our sensitivity analysis (**Table 1**), P_tot_ (*initial pMHC MDC)*, a parameter combining multiple terms representing DC activation and amount of stimulation T cells receive, was significantly correlated with size of both CD4+ and CD8+ T cell memory populations, but the sign and strength of correlation changed with binding probability. For both CD4+ and CD8+ T cells with high binding probability (Ag1, Ag2), P_tot_ was negatively correlated to memory population size, whereas at low binding probabilities (Ag4, Ag5), P_tot_ was positively correlated with size of memory T cell populations in Memory Design Space.

If the effects of changing P_tot_ vary across Ag-specific populations, we reasoned, then there must be some LN environmental conditions (specific P_tot_ levels) for which binding probability affects more than the total number of cells generated. We show results from CD8+ T cells here, but CD4+ T cells had similar patterns. We plotted numbers of each memory subtype generated from LNs with low, medium and high P_tot_ for T cells with high binding probability (**Figure 3C**, left). Increasing P_tot_ correlated with a decreased number of both CM and EM T cells in blood. This is again related to amount of stimulation cells receive while bound: stronger activated DCs (with higher P_tot_) in our model pass on stronger stimulation to their bound T cells (Gett and Hodgkin, 2000). In the corresponding Memory Design Space interpretation, cells from LN environmental conditions that have the lowest P_tot_ generated the largest total number of memory cells, driven by a large CM T cell population, and as P_tot_ increased, size in Memory Design Space decreased.

We then plotted the memory CD8+ T cells from medium (**Figure 3C**, middle) and low (**Figure 3C**, right) binding probability populations produced from the same LN environment, under the same Ag-independent conditions. With low P_i_(bind), we noticed the opposite trend: increased activation correlated with increased numbers of memory cells generated, especially EM T cells. In Memory Design Space, this translated to populations that move up along the y-axis, each with a different size and skew. This occurred as P_tot_ has a secondary effect: DCs with high P_tot_ display more antigen and thus yield higher P_i_(bind) for all Ag-specific T cells than their lower P_tot_ counterparts. Therefore, for Ag-specific T cells with low binding probability, increasing P_tot_ increased the number of cells that bind, and for theses cells this effect was stronger than the difference in CM versus EM T cell differentiation that we observed for populations with higher binding probabilities.

At the highest level of P_tot_ tested, we found that Ag-specific populations did not have the same EM/CM ratio (**Figure 3D**). Binding probability still correlated with size, but for lower binding probability populations, there was a slight skew toward EM T cells and thus a higher EM/CM ratio (i.e., greater skew in Memory Design Space). As we observed in **Figure 3A**, a single priming event using our model was unable to generate the full range of EM/CM ratios with populations of equal sizes. To cover more of Memory Design Space, we needed to increase the size of EM-skewed populations, so we considered how additional vaccinations via boosting.

### Boosting Once Increases the Size of the Memory T Cell Population; Boosting Twice Additionally Changes the Skew

**FIGURE 4:**
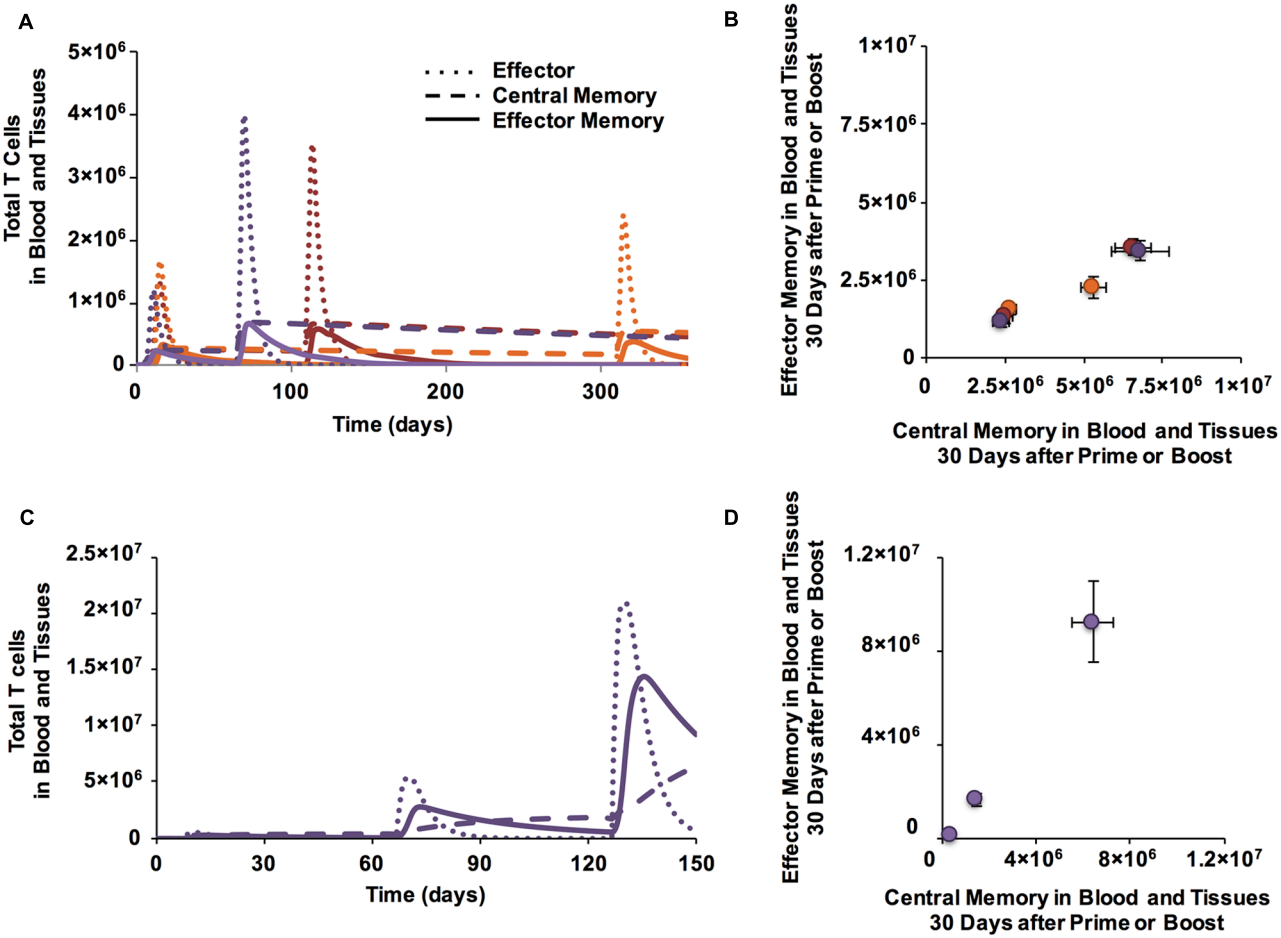
**Boosting increases the size of memory populations and can shift EM/CM ratios. (A)** Time courses of CD4+ T cells in blood and peripheral tissues after prime and boost (purple: boost at day 60, red: boost at day 100, orange: boost at day 300). **(B)** Memory populations corresponding to time series from Panel A are plotted in Memory Design Space. CD4+ T cells in blood and peripheral tissues are counted at 30 days following each prime or boost. Error bars indicate SEM (*n* = 10). **(C)** Additional boosts increase number of CD8+ CM and EM T cells generated over time. **(D)** A second boost increases EM/CM ratio. Memory populations corresponding to time series from C and measured 30 days following a prime and two boosts are plotted in Memory Design Space. Error bars indicate SEM (n = 10). See Supplementary Figure S2 for analysis of change in skew.

Many vaccines require more than one dose to reach their full potential (Woodland, 2004). Several studies have shown that additional stimulation events (boosting) can increase the number of memory T cells, enhance their avidity, and even change the memory subtypes that are generated (Busch and Pamer, 1999; Masopust et al., 2006; Wirth et al., 2010; Khanolkar et al., 2013; Martin and Badovinac, 2014). Therefore, we investigated the effects of a second stimulation (boost) event on memory T cell generation, i.e., location in Memory Design Space. The boost was given at 60, 100, or 300 days after priming, with the same stimulus and conditions as the first stimulation. In agreement with literature (Woodland, 2004) and previous versions of our model (Gong et al., 2014), a boost at 60 days generated more effector CD4+ T cells and more memory T cells than the initial stimulation (**Figure 4A**). This translated to a greater memory population size, but no significant change in EM/CM ratio (**Figure 4B**). CD8+ T cells showed similar dynamics, but we only show CD4+ T cells here. There is little difference between boosting at 60 or 100 days, but a decrease in the total number of memory cells generated after a much later boost (day 300). This is expected because the number of memory cells remaining after the prime decreases slowly over time. Boosts result in a change in memory population size but not skew (**Figure 4B**).

To control how much stimulation a T cell receives independent from other Ag-specific populations, the most direct way is to control the number of stimulation events each population receives. Therefore, we simulated a time course of three stimulation events: a prime followed by a boost 60 days later and a second boost 60 days after that (**Figure 4C**). After the second boost, the population shifts up the y-axis in Memory Design Space (**Figure 4D**), indicating a large number of newly generated EM T cells. This result agrees with experimental results (Masopust et al., 2006) wherein increasing the number of stimulation events caused memory T cell populations that had been stimulated to skew toward EM. Comparisons of skew of memory populations following each stimulation event are shown in Supplementary **Figure 2**.

### A Full Range of EM/CM Ratios and Memory Population Sizes can be Achieved by Varying Binding Probability, DC Activation, and Number of Vaccine Doses

**FIGURE 5:**
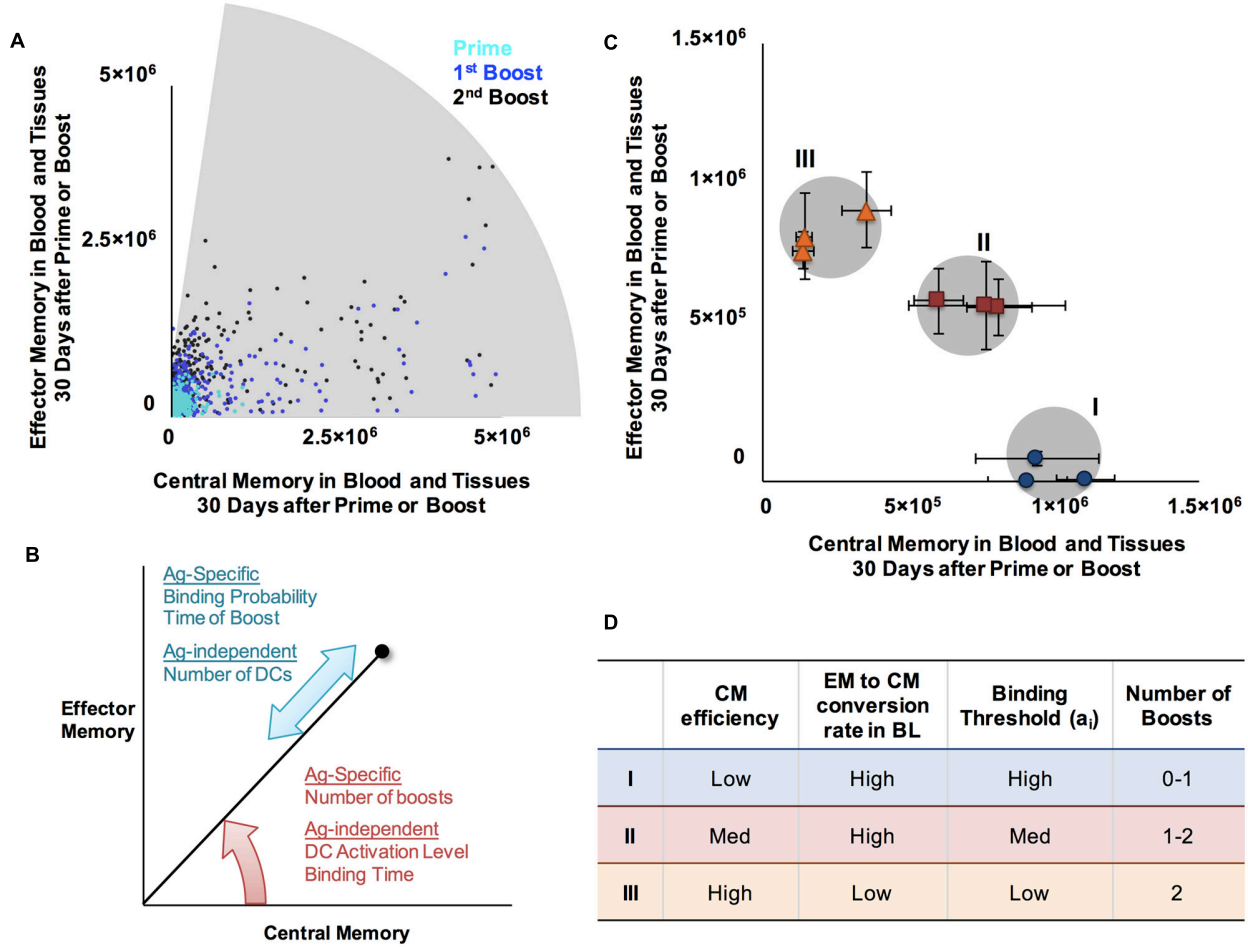
**A range of CM/EM ratios and memory population sizes can be reached following prime and boost. (A)** Shaded region represents EM/CM ratios and memory population sizes achieved at the memory time point, 30 days after priming event (cyan, same data as **Figure 3A**), prime and boost (blue), or prime and two boosts (black) for CD4+ memory T cells. **(B)** Summary of mechanisms affecting EM/CM ratio (red arrow) and memory population size (blue arrow) as determined by sensitivity analysis. **(C)** Generating memory populations with specific sizes and EM/CM ratios (skew). We identified three desired memory populations (I, II, and III and gray circles). We then designed vaccination simulations to generate these desired memory populations ratios and sizes. Simulated T cell populations representing the closest match for each desired memory population are plotted in Memory Design Space, on the same axes for comparison: blue dots (desired population I), red squares (population II), and orange triangles (population III). Error bars represent SEM (*n* = 10). **(D)** Summary of approximate parameter values to achieve specified positions in Memory Design Space. The Ag-specific and Ag-independent parameters noted here were varied simultaneously to achieve desired populations. The ranges over which each parameter was varied are as follows: CM efficiency 0.1-10, EM to CM conversion in BL 0-0.188 (baseline value), binding threshold: 30-300, number of boosts: 0-2. For each of the desired memory population, three independent parameter sets that generated populations with desired characteristics were identified. Exact parameter values are given in Supplementary Table 2.

To determine the range of EM/CM ratios and sizes of memory populations that the model is able to generate following boosting events, we performed simulations over a range of Ag-independent and Ag-specific parameters, and plotted the resulting populations in Memory Design Space (**Figure 5A**). For comparison to **Figure 3A**, we again show CD8+ populations; CD4+ T cells followed similar patterns. As we observed earlier, a single boost is able to increase the number of cells produced (especially if given within a few months after prime), but not the proportion of EM cells remaining after prime. A second boost, however, not only further increases the number of memory cells, but also shifts the population toward EM. These mechanisms, coupled with the Ag-independent and Ag-specific mechanisms that determine the amount and type of memory cells generated after prime allow us to reach most regions of Memory Design Space up to very high numbers of Memory T cells. The model mechanisms that govern position in Memory Design Space are summarized in **Figure 5B**.

To design a vaccine, one might desire to produce a memory T cell population with a specific size and skew. To demonstrate this concept, we chose three desired points in Memory Design Space and used our model to generate Ag-specific T cell populations near those points. To simulate a vaccine intended to prime Ag-specific T cell populations with distinct memory characteristics, we chose the set of points to vary in skew in Memory Design Space have population sizes greater than 10^6^. In **Figure 5C**, the shaded regions in Memory Design Space represent desired memory populations. For each scenario, we plot three memory CD4+ T cell populations with distinct EM/CM ratios and sizes within an acceptable range of the target population size. We found that very similar parameter combinations worked for CD4+ and CD8+ populations, but CD8+ populations reached higher numbers due to more rounds of proliferation. **Figure 5D** notes the parameter values and vaccination schedules that we used to reach the desired memory population sizes and skew, and the memory populations generated by the model using the stated parameters.

### In a Simulated *M. tuberculosis* Infection, Two Ag-Specific Memory T Gell Populations can Prevent Granuloma Establishment or Protect Against Reactivation

Protection from TB can be assessed by asking whether a vaccine improves clearance rates from a new infection and/or whether it reduces the probability of a latent infection reactivation relative to an unvaccinated case. One recently promising TB vaccine is H56, a multi-subunit vaccine containing antigens specific to both early and later phases of infection (Aagaard et al., 2011). Non-human primates (NHPs) vaccinated with H56 did not progress to active disease after infection with *M. tuberculosis;* all either cleared the infection or contained the bacilli within granulomas (Lin et al., 2012). Furthermore, when the H56-vaccinated NHPs were given an anti-TNF antibody known to induce reactivation (Adalimumab), a significant proportion (50%) were protected and did not reactivate (Lin et al., 2012). H56 is a subunit vaccine containing five antigens, one of which is associated with late phases of infection (Aagaard et al., 2011) and correlated with shifted *M. tuberculosis* metabolism (Voskuil et al., 2004), while some other antigens in H56 are associated with earlier stages (Aagaard et al., 2011).

**FIGURE 6:**
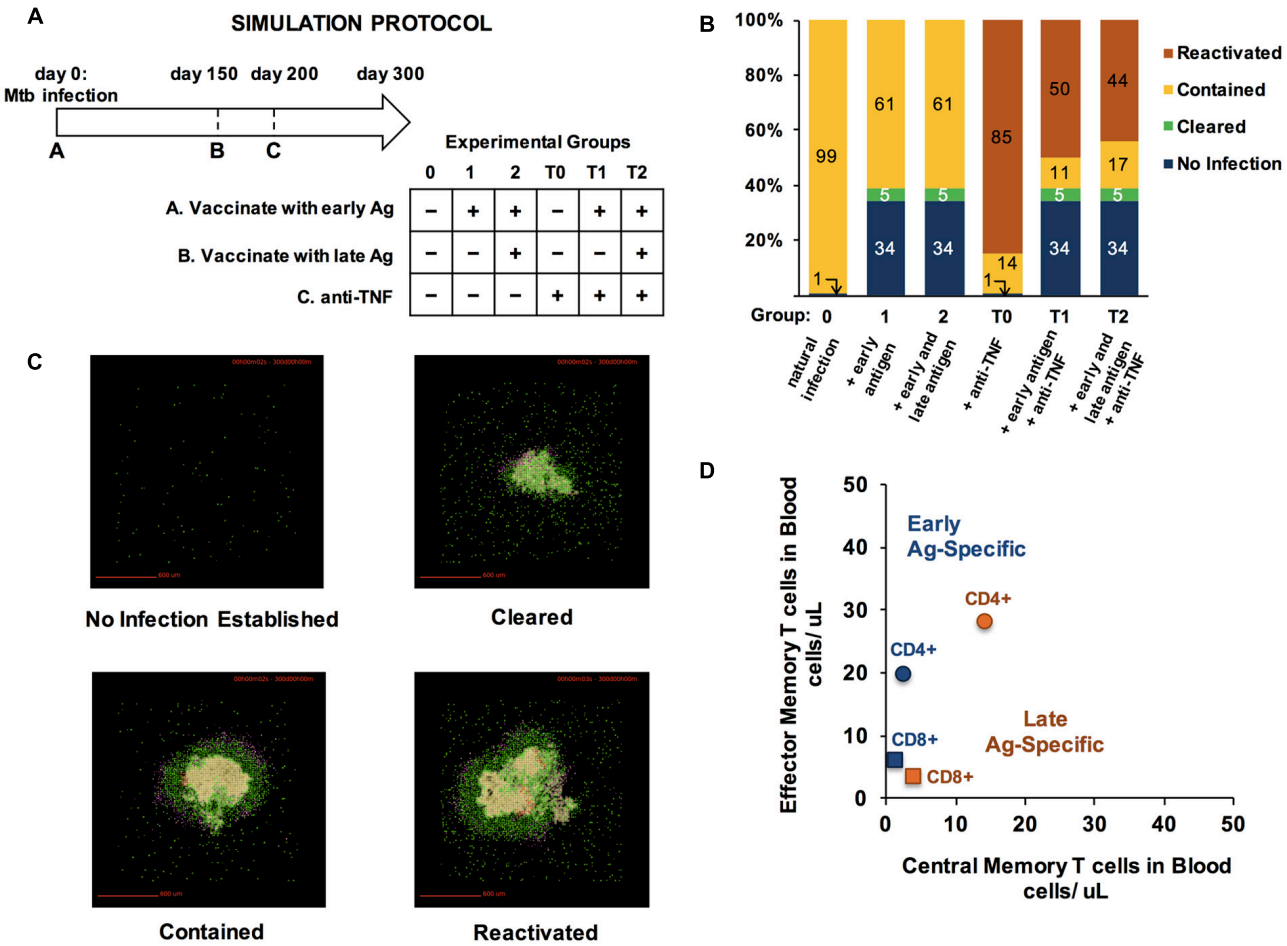
**Vaccinating with early and late-infection stage specific antigens improves protection from TB infection and prevents reactivation of contained granulomas. (A)** Simulation Protocol. Four groups received a combination of three treatments over a 300-day experiment. All experimental groups were infected with *M. tuberculosis* on day 0. Prior to infection, groups 1, 2, T1, and T2 received T cells specific to early infection antigens on day 0. On day 150, groups 2 and T2 received an additional bolus of T cells, simulating specificity to late antigens. Anti-TNF treatments were administered to groups T0, T1, and T2 on day 200 (hence the T label). All outcomes were scored on day 300 post-infection. **(B)** Simulation results for each group (100 simulations each). Outcomes are defined as described in section “Materials and Methods.” **(C)** Representative snapshots from day 300 p.i. from granuloma model simulations. Colors represent cell types. Green: resting macrophages, blue: active macrophages, red: chronically infected macrophages, tan: infected macrophages, pink: IFN-γ-producing T cells, purple: cytotoxic T cells, pink: regulatory T cells, beige/cream: caseated/ necrotic tissue. **(D)** To achieve the benefits of vaccination observed in panel B, we used uncertainty analyses and tested 50 configurations of memory populations specific to early and late TB antigens. The number of CD4+ and CD8+ EM and CM T cells administered as a vaccine at either day 0 (“Early Ag-Specific”) or day 150 (“Late Ag-Specific”) that yielded the results from **(B)** are plotted in Memory Design Space in **(D)**.

To assess the effects of different Ag-specific memory T cell populations as a vaccine strategy, we designed a virtual vaccine-challenge experiment using our existing three-compartment model of *M. tuberculosis* infection at the granuloma scale, *GranSim*, which includes lung, blood, and LN physiological compartments (Linderman et al., 2015). Since the sites of infection for TB are lung granulomas, and vaccination usually is administered in blood, we place representative memory T cells specific to early and late phase TB antigens in the blood when we are administering a virtual vaccine (simulation protocol in **Figure 6A**). These T cells are available to be recruited to a granuloma site much sooner than in a naïve system in which Ag presentation must drive priming in the LN, followed by migration back to the granuloma site. We explored multiple configurations of memory populations in the blood by varying the amount of EM and CM for CD4+ and CD8+ T cell populations specific to both early and late antigens. We then initiated infection in the lungs by introducing *M. tuberculosis* at time zero. We also simulated a typical *in vivo* protocol for inducing reactivation, treating the virtual granuloma with anti-TNF antibodies. This leads to reactivation in a NHP model of latent TB (Lin et al., 2012) and allows us to test whether the granuloma that forms is stable, i.e. able to maintain control of infection or whether it progresses to dissemination (growing larger in both size and bacterial load).

For each protocol, we assessed granuloma outcomes at day 300 after a simulated infection as described in Methods. Six simulated groups, each of 100 granulomas and infected with *M. tuberculosis* at day 0, were examined (**Figure 6A**). Group 0 received no vaccine and no anti-TNF. Group T0 received no vaccine but was treated with anti-TNF on day 200 p.i. Group T1 received a vaccine containing only T cells specific to early phases of infection at the same time as *M. tuberculosis* infection on day 0, followed by anti-TNF on day 200. Group T2 received a vaccine with T cells populations specific to antigens in both early and latency phases of infection concurrent with *M. tuberculosis* challenge on day 0 and anti-TNF on day 200. Groups 1 and 2 received the same vaccines as Groups T1 and T2, respectively, but did not receive anti-TNF.

With no virtual vaccination or anti-TNF treatment, *M. tuberculosis* infections were contained in 99% of cases, and in one case out of 100, did not establish infection (Group 0, **Figure 6B**). Vaccinating with either an early antigen alone (Group 1) or both early and late antigens (Group 2) prevented infection from establishing in 34% of simulations and caused 5% of granulomas to clear. Compared to Group 0, adding anti-TNF antibodies at day 150 to induce reactivation (Group T0) increased the number of disseminating granulomas from 0 to 85%. Granuloma group T1, which was vaccinated with an antigen that is specific to early stage TB infection and given anti-TNF antibodies, had 50% of granulomas disseminate, all of which were contained when anti-TNF was spared (Group 1). In Group T2, which was vaccinated with both early and late antigens and treated with anti-TNF, fewer granulomas reactivated (44%), suggesting that presence of memory T cells specific to late-stage Ag is beneficial to protect from reactivation of a stable granuloma to a disseminating one. Example granuloma simulation snapshots at day 300 are shown in **Figure 6C**.

The size and skew of CD4+ and CD8+ memory T cell populations used in our virtual vaccine trial (**Figures 6A,B**) are plotted in **Figure 6D**. These choices were determined via uncertainty and sensitivity analyses over 50 parameter combinations to identify concentrations of each set of TB-specific CM and EM T cells that would give tangible benefits in both controlling infection and preventing reactivation (**Figure 6B**). It is notable that each Ag-specific population has a unique skew and size, with the early specific populations having a larger skew than late-specific memory T cells, and CD4+ populations much larger than CD8+. It is precisely this design strategy that can be used to design vaccine outcomes for complex infections such as TB. Here we reverse-engineered desired vaccine outcomes by screening for successful combinations of T cell concentrations. However, *GranSim* and *LymphSim* are independent of each other, suggesting that it is possible to identify desired regions in Memory Design Space, design vaccines to reach those regions, and demonstrate the effect of the vaccine in a virtual vaccine trial, at least at the granuloma level.

## Discussion

Historically, it has been difficult to create vaccines that generate cell-mediated immunity, and attention has focused instead on antibody-mediated responses. Despite modern advances to induce large and long-lasting populations of antigen-specific memory T cells, there is no efficacious vaccine against *M. tuberculosis* or several other infections requiring cell-mediated immunity. Furthermore, especially for TB, it is becoming increasingly clear that quality and specificity of those cells is just as important of a feature as quantity of memory T cells. The TB Vaccine Initiative has identified top priorities in adopting a more rational approach to vaccine design (TBVI, 2014), and the recent goals and achievements cited by Aeras (Aeras, 2014), an independent organization that also develops TB vaccines, are aligned. Those priorities include novel immunization strategies, optimization of immunogenicity, and identifying a model with great relevance to clinical efficacy.

Our work facilitates the next step in rational vaccine design. But it must be clear that this virtual vaccine study examines only events at a single granuloma scale and much work needs to be done to extend this to results at the whole host. Our computational model represents a framework in which new vaccination protocols, delivery routes, and other strategies can easily be tested and compared at a granuloma scale. Mechanisms that are key to protection can be identified, and the consequences of modulating vaccine properties can be examined. Thus computational modeling, while not replacing *in vivo* studies, offers a complementary tool that allows integration of knowledge from *in vitro, ex vivo*, and *in vivo* experiments across multiple organ systems to help narrow the vaccine design space. This work must be extended now to the entire host where many other factors such as adjuvants, multiple antigens, multiple LNs, and their locations each add to the complexity. With a much larger possible number of replications, simulated vaccine trials could be used to bolster results from animal studies with low power by recapitulating and explaining seemingly anomalous outcomes that actually represent significant results. Furthermore, simulated vaccine trials could represent cost and time savings by ferreting out strategies that have otherwise unforeseen pitfalls, e.g., a recent group of failed vaccine trials that were derived solely from mice studies (Tameris et al., 2013; Hawn et al., 2014; Kaufmann, 2014).

One frustration in identifying correlates of protection against TB is that it is not currently well-understood whether the protective Ag-specific memory T cell populations are being identified and thus targeted, or, as many suspect, whether some functional property of memory populations needs to be modulated. Memory Design Space is a framework to separate these two issues visually. Each Ag-specific population may confer protection from a different region of Memory Design Space. If, as we hypothesize, the EM/CM ratio of memory T cells generated is crucial to protection, especially if the crucial ratio varies across multiple Ag-specific populations required for protection, our computational model of the lymph node, *LymphSim*, could be used to design a vaccination strategy that would strategically produce the desired memory populations in the appropriate quantity, with the right EM/CM skews.

Using *LymphSim*, we showed how both Ag-specific and Ag-independent properties influence location in Memory Design Space. Notably, Ag-specific properties in our model all influenced the amount of memory cells generated, but not the type (or their ratio). Ag-independent properties, on the other hand, had a stronger effect on the types of memory generated rather than the amounts. Antigens with identical characteristics that were involved in priming in LNs with different Ag-independent properties yielded memory T cell populations of similar sizes but different memory subtype composition. However, after a single round of vaccination (priming) the range of EM/CM ratios was still narrow. Providing additional rounds of vaccination (repeated boosts) achieved a full range of EM/CM ratios. In agreement with the literature, increasing the number of boosts increased the EM proportion of the memory populations. We also showed that by combining strategies that modify Ag-specific and Ag-independent properties in combination with vaccine delivery schedules we are able to generate memory T cell populations specific to multiple antigens with a large variety of EM/CM ratios and population sizes.

As a preliminary test of our vaccine system, we considered a two-antigen system for TB. Using our computational model of *M. tuberculosis* infection in the lung (*GranSim)*, we demonstrated how T cell populations generated from vaccination could be evaluated in an infection scenario at the level of a single granuloma. We introduced two populations of memory T cells into the blood compartment of the infection model, as if they had been conferred by a vaccine. The first population was specific to an antigen present during early stages of infection and granuloma formation whereas the second was specific to late phases of infection, when bacterial metabolism has shifted. We showed that presence of both populations of memory T cells with specific subtype compositions led to better granuloma outcomes than with one population. We identified the positions of each T cell population in Memory Design Space that led to beneficial outcomes, and showed earlier how we could use our LN computational model to design a vaccine that could simultaneously generate all of the populations used in this study. In future studies, exploration of host-level outcomes (multiple granulomas are present in a single host (Marino et al., under review) and the role of adjuvants will be important. Thus, we have provided a road map for using Memory Design Space to characterize T cell populations resulting from vaccines and then relating outcomes of vaccination back to position in Memory Design Space. This provides an objective comparison across both inputs and outputs of a virtual vaccine trial.

In a more comprehensive study, our model could be used to find regions of Memory Design Space representing memory T cell populations that confer protection across a wide variety of simulated granulomas that will allow extension to the whole host disease picture. Validation of this work could be accomplished using NHP studies after model parameters governing timing of Ag expression, processing, and display were experimentally measured and calibrated to measurements of the granulomas and then scaling to whole host outcomes in some fashion. Furthermore, the results of such a study could elucidate which model mechanisms make the best targets for preventing infection, contributing to our understanding of correlates of protection against TB. EM/CM ratio, stage of infection targeted, amount of cytokines produced, and several other mechanisms could prove to be (or not be) important. Our model framework provides an advantage over *in vivo* or *in vitro* experiments since it is inexpensive to run replicates of almost infinite combinations of T cells; for several antigens simultaneously allowing a narrowing of the vaccine design space for going to trials.

## Author Contributions

CZ and CG conceived of model changes, coded model, and designed experiments. CZ carried out experiments, analyzed data, and wrote the paper. DK and JL consulted on model changes, experimental design, and analysis; and edited the paper.

## Conflict of Interest Statement

The authors declare that the research was conducted in the absence of any commercial or financial relationships that could be construed as a potential conflict of interest.
