# Supplementary material for: Strategic Priming with Multiple Antigens can Yield Memory Cell Phenotypes Optimized for Infection with Mycobacterium tuberculosis: A Computational Study
Source: Front Microbiol. 2016 Jan 6;6:1477. doi: 10.3389/fmicb.2015.01477 (PMC4701940; doi:10.3389/fmicb.2015.01477)
Supplement: Supplementary file 1 [file Data_Sheet_1.DOCX]

Supplementary Material

**Strategic Priming with Multiple Antigens Can Yield Memory Cell Phenotypes Optimized for Infection with *Mycobacterium tuberculosis***

**Cordelia Ziraldo, Chang Gong, Denise E Kirschner, Jennifer J Linderman***

*** Correspondence:** Jennifer Linderman: linderma@umich.edu

# Model Rules

All model rules can be found at: <http://malthus.micro.med.umich.edu/lab/movies>. The rules are exactly as described in Gong et al., 2014, except for the following additions:

An additional set of ODEs was incorporated to track cells migrating from blood to peripheral tissues and; the blood equations remain exactly the same as in Gong 2014. The new peripheral tissues equations are given in Methods.

All Blood/ Peripheral Tissue ODE equations were copied so that each Ag-specific population could evolve independently in these blood and peripheral tissues. In this work, we did not vary ODE parameters between Ag-specific sets of equations, though we had the capability. This was so we could better understand the events in the LN, not in the blood or tissues, which is left for other work.

For CM T cells in the LN: In Gong 2014, if there is a mature or licensed DC within the binding radius, the CM cells bind and switch to Bound state. Here, before CM binding occurs additional criteria must be met:

1. the CM cell cannot be newly generated. Only CM cells that have entered the LN from blood (rather than differentiating after unbinding from a DC) are eligible to bind.
2. the DC must be displaying cognate p_i_MHC

# Supplementary Figures and Tables

## Supplementary Figures


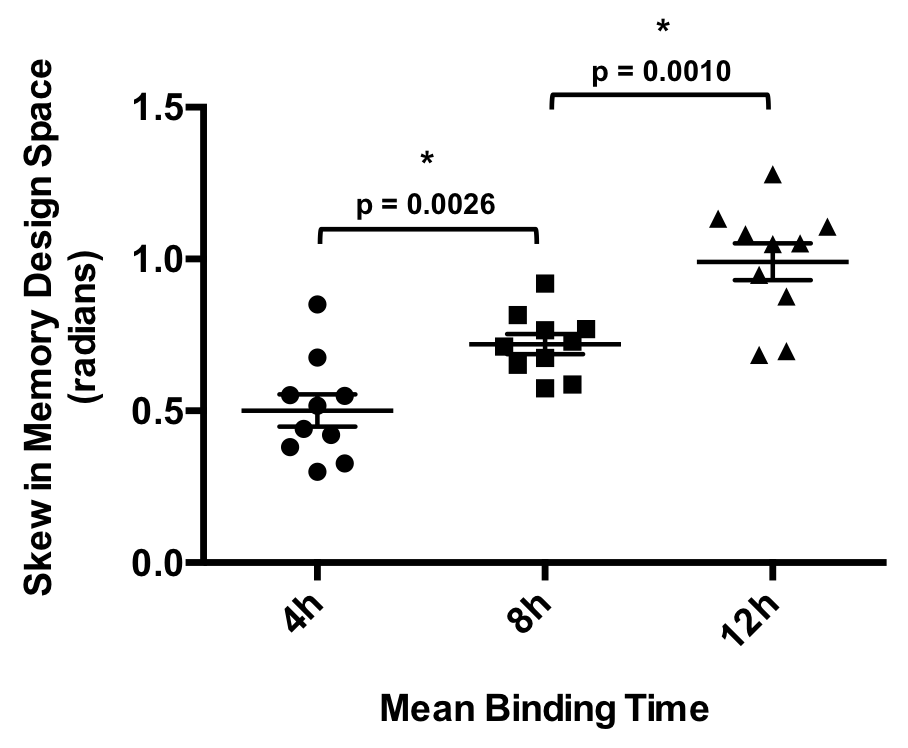


**Supplementary Figure 1.** Skew of Memory Populations measured 30 days after a prime. Each point in Fig 2E is the mean of 10 replicates; each replicate has its own location in Memory Design Space, with associated skew and distance. Here we plot the skew of all replicates for each of 3 points, each with different average DC-T cell binding time during priming. Lines indicate mean ± SEM. p-values for significance calculated using unpaired t-tests, assuming Gaussian distribution and equal SD.


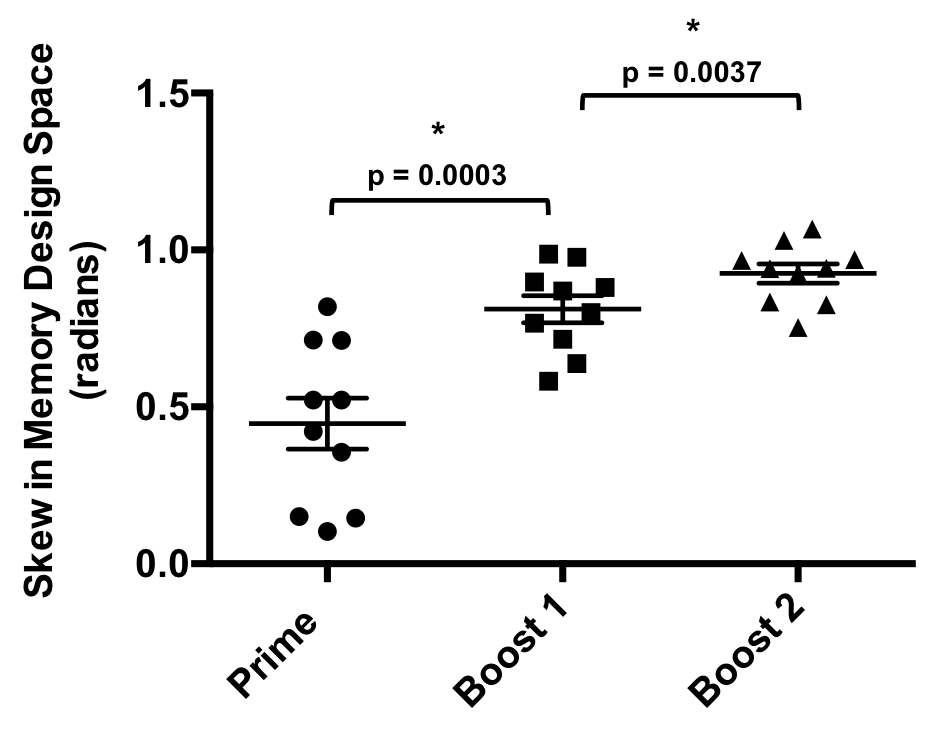


**Supplementary Figure 2.** Skew of Memory Populations measured 30 days after a stimulation event (prime or boost). We plot the skew of 10 replicates for each of 3 points from Fig 4D, representing prime and 2 subsequent boosts. Lines indicate mean ± SEM. p-values for significance calculated using paired t-tests, assuming Gaussian distributions and equal SDs.

## Supplementary Tables

**Supplementary Table 1. Baseline parameter values.** Listed here are all parameters that can be varied in the *LymphSim* agent-based model (ABM) and the associated ODEs representing blood and peripheral tissues (“non-lymphatic tissues”, NLT). For brevity we list only one instance of each Ag-specific parameter, but for each antigen there is an individual parameter whose value can be varied independently.

| PARAMETER | **AG RELATION** | **BASELINE VALUE (LHS RANGE)** | **UNITS** | | | **REFS** |
| --- | --- | --- | --- | --- | --- | --- |
| Architecture and Cell Movement |  |  |  | | |  |
| run to steady state interval | Ag-independent | 6912 | LN ABM time step | | |  |
| LN to blood scaling factor | Ag-independent | 200 | none | | |  |
| Simulated time per ABM time step | Ag-independent | 25 | seconds | | |  |
| HEV count | Ag-independent | 180 | count | | |  |
| HEV top slice | Ag-independent | 154 | LN ABM grid space | | |  |
| HEV bottom slice | Ag-independent | 50 | LN ABM grid space | | |  |
| EL count | Ag-independent | 300 | count | | |  |
| EL ydelta | Ag-independent | 10 | LN ABM grid space | | |  |
| EL top slice | Ag-independent | 77 | LN ABM grid space | | |  |
| prob move in same direction | Ag-independent | 0.85 | LN ABM time step^-1^ | | |  |
| prob move in non-opposite direction | Ag-independent | 0.95 | LN ABM time step^-1^ | | |  |
| prob move in same direction block | Ag-independent | 0.6 | LN ABM time step^-1^ | | |  |
| prob move in non-opposite direction block | Ag-independent | 0.92 | LN ABM time step^-1^ | | |  |
| binding radius | Ag-independent | 2 | LN ABM grid space | | | ([Miller et al., 2004b](#_ENREF_19)) |
| relocation radius | Ag-independent | 2 | LN ABM grid space | | |  |
| unbinding check frequency | Ag-independent | 1 | LN ABM time step | | |  |
|  |  |  |  | | |  |
| CD4+ T cells |  |  |  | | |  |
| min age | Ag-independent | 165 | days | | | ([McCune et al., 2000](#_ENREF_17)) |
| max age | Ag-independent | 365 | days | | |  |
| max age std dev | Ag-independent | 15 | days | | |  |
| avg lifespan Naïve | Ag-independent | 365 | days |  | | |
| max binding time Naïve | Ag-independent | 8 | hours |  | | |
| max binding time Naïve std dev | Ag-independent | 4 | hours |  | | |
| max binding time CM | Ag-independent | 45 (20-60) | minutes |  | | |
| max binding time CM std dev | Ag-independent | 10 | minutes |  | | |
| median priming prob | Ag-independent | 1000  (500-2000) | Accumulated stimulation | | | |
| shape priming prob | Ag-independent | 100 | Accumulated stimulation | | | |
| max lifetime Active | Ag-independent | 96 | hours |  | | |
| max lifetime Active std dev | Ag-independent | 4 | hours |  | | |
| avg lifetime Active | Ag-independent | 96 | hours |  | | |
| min divisions | Ag-independent | 6 | count | ([Foulds et al., 2002](#_ENREF_9);[Miller et al., 2004a](#_ENREF_18)) | | |
| max divisions | Ag-independent | 12 | count |  | | |
| doubling period | Ag-independent | 6 | hours | ([Linderman et al., 2010](#_ENREF_15)) | | |
| median effector prob | Ag-independent | 2000  (1500-3000) | Accumulated stimulation | | | |
| shape effector prob | Ag-independent | 500 | Accumulated stimulation | | | |
| max lifetime Effector | Ag-independent | 60 | hours | ([Sprent and Tough, 2001](#_ENREF_23)) | | |
| max lifetime Effector std dev | Ag-independent | 1 | hours |  | | |
| avg lifetime Effector | Ag-independent | 60 | hours |  | | |
| max lifetime CM | Ag-independent | 365 | days |  | | |
| max lifetime CM std dev | Ag-independent | 15 | days |  | | |
| avg lifetime CM | Ag-independent | 365 | days |  | | |
| max lifetime EM | Ag-independent | 365 | days |  | | |
| max lifetime EM std dev | Ag-independent | 15 | days |  | | |
| avg lifetime EM | Ag-independent | 25 | days |  | | |
| begin searching for DCs | Ag-independent | 3 | days |  | | |
| probability of recruitment | Ag-independent | 0.26 | LN ABM time step^-1^ | |  | |
| extra recruitment | Ag-independent | 1.25 (1-1.5) | none | |  | |
| efficiency Central Memory | Ag-independent | 6 (0.1-10) | hours^-1^ | |  | |
| prob Effector Memory | Ag-independent | 0.1  (0.025-0.4) | LN ABM time step^-1^ | |  | |
| prob initialize Naive | Ag-independent | 0.422 | LN ABM time step^-1^ | | |  |
| Ag-specific Cognate Frequency | Ag-specific | 1.00E-04 | cells^-1^ | | | ([Blattman et al., 2002](#_ENREF_3);[Moon et al., 2007](#_ENREF_20)); ([Casrouge et al., 2000](#_ENREF_4)) |
| Ag-specific binding threshold (a_i_) | Ag-specific | 150 | pMHC | | | ([Demotz et al., 1990](#_ENREF_7)) |
| Ag-specific binding slope (b_i_) | Ag-specific | 15 | pMHC | | |  |
| pMHC-II Fraction (m_i_) | Ag-specific | 0.2 | none | | |  |
|  |  |  |  | | |  |
| CD8+ T cells |  |  |  | | |  |
| min age | Ag-independent | 165 | days | | |  |
| max age | Ag-independent | 365 | days | | |  |
| max age std dev | Ag-independent | 15 | days | | |  |
| avg lifespan Naïve | Ag-independent | 365 | days | | |  |
| max binding time Naïve | Ag-independent | 8 | hours | | |  |
| max binding time Naïve std dev | Ag-independent | 4 | hours | | |  |
| max binding time CM | Ag-independent | 45 (20-60) | minutes | | |  |
| max binding time CM std dev | Ag-independent | 10 | minutes | | |  |
| median priming prob | Ag-independent | 1000  (500-2000) | Accumulated stimulation | | | |
| shape priming prob | Ag-independent | 100 | Accumulated stimulation | | | |
| max lifetime Active | Ag-independent | 96 | hours | | |  |
| max lifetime Active std dev | Ag-independent | 4 | hours | | |  |
| avg lifetime Active | Ag-independent | 96 | hours | | |  |
| min divisions | Ag-independent | 8 | count | | | ([Foulds et al., 2002](#_ENREF_9);[Miller et al., 2004a](#_ENREF_18)) |
| max divisions | Ag-independent | 20 | count | | |  |
| doubling period | Ag-independent | 6 | hours | | | ([Linderman et al., 2010](#_ENREF_15)) |
| median effector prob | Ag-independent | 2000  (1500-3000) | Accumulated stimulation | | | |
| shape effector prob | Ag-independent | 500 | Accumulated stimulation | | | |
| max lifetime Effector | Ag-independent | 60 | hours | | | ([Sprent and Tough, 2001](#_ENREF_23)) |
| max lifetime Effector std dev | Ag-independent | 1 | hours | | |  |
| avg lifetime Effector | Ag-independent | 60 | hours | | |  |
| max lifetime CM | Ag-independent | 365 | days | | |  |
| max lifetime CM std dev | Ag-independent | 15 | days | | |  |
| avg lifetime CM | Ag-independent | 365 | days | | |  |
| max lifetime EM | Ag-independent | 365 | days | | |  |
| max lifetime EM std dev | Ag-independent | 15 | days | | |  |
| avg lifetime EM | Ag-independent | 60 | days | | |  |
| begin searching for DCs | Ag-independent | 3 | days | | |  |
| probability of recruitment | Ag-independent | 0.13 | LN ABM time step^-1^ | | |  |
| extra recruitment | Ag-independent | 1.25 (1-1.5) | none | | |  |
| efficiency Central Memory | Ag-independent | 6 (0.1-10) | hours^-1^ | | |  |
| prob Effector Memory | Ag-independent | 0.1  (0.025-0.4) | LN ABM time step^-1^ | | |  |
| prob initialize Naive | Ag-independent | 0.211 | LN ABM time step^-1^ | | |  |
| Ag-specific Cognate Frequency | Ag-specific | 1.00E-04 | cells^-1^ | | | ([Blattman et al., 2002](#_ENREF_3);[Obar et al., 2008](#_ENREF_21)) |
| Ag-specific binding threshold (a_i_) | Ag-specific | 150 | pMHC | | | ([Henrickson et al., 2008](#_ENREF_10)) |
| Ag-specific binding slope (b_i_) | Ag-specific | 15 | pMHC | | |  |
| pMHC-I Fraction (m_i_) | Ag-specific | 0.2 | none | | |  |
| Dendritic Cells |  |  |  | | |  |
| min age | Ag-independent | 1 | days | | |  |
| max age | Ag-independent | 11 | days | | |  |
| max age std dev | Ag-independent | 2 | days | | |  |
| avg lifespan IDC | Ag-independent | 5 | days | | | ([Kamath et al., 2002](#_ENREF_13)) |
| initial pMHC IDC | Ag-independent | 50 | pMHC | | |  |
| initial pMHC IDC std dev | Ag-independent | 50 | pMHC | | |  |
| IDC maturation increment pMHC | Ag-independent | 200 | pMHC | | |  |
| IDC maturation increment pMHC std dev | Ag-independent | 150 | pMHC | | |  |
| ratio MDC recruited | Ag-independent | 0.6 | LN ABM time step^-1^ | | |  |
| initial pMHC MDC (P_tot_) | Ag-independent | 300 (50-500) | pMHC | | |  |
| initial pMHC MDC std dev | Ag-independent | 50 | pMHC | | |  |
| pMHC-I half life | Ag-independent | 25 | hours | | | ([Cella et al., 1997](#_ENREF_5)) |
| pMHC-II half life | Ag-independent | 100 | hours | | | ([Cella et al., 1997](#_ENREF_5)) |
| unbinding threshold | Ag-independent | 50 | pMHC | | |  |
| avg age entry MDC | Ag-independent | 20 | hours | | |  |
| max lifetime MDC | Ag-independent | 60 | hours | | | ([Kamath et al., 2002](#_ENREF_13)) |
| max lifetime MDC std dev | Ag-independent | 5 | hours | | |  |
| avg lifespan MDC | Ag-independent | 60 | hours | | |  |
| prob CD4+ Eff licences MDC | Ag-independent | 0.005  (0.001-0.1) | LN ABM time step^-1^ | | |  |
| time being LDC | Ag-independent | 36 | hours | | | ([Lanzavecchia and Sallusto, 2004](#_ENREF_14);[Lindquist et al., 2004](#_ENREF_16)) |
| time being LDC std dev | Ag-independent | 4 | hours | | |  |
| avg lifespan LDC | Ag-independent | 36 | hours | | |  |
| probability of recruitment to LN | Ag-independent | 0.03 | LN ABM time step^-1^ | | |  |
| probability of initialization to LN | Ag-independent | 0.0006 | LN ABM time step^-1^ | | |  |
| prob IDC activation | Ag-independent | 0.01 | LN ABM time step^-1^ | | |  |
| movement interval | Ag-independent | 4 | LN ABM time step | | | ([Miller et al., 2004a](#_ENREF_18)) |
| recruitment start | Ag-independent | 3 | days | | |  |
| recruitment end | Ag-independent | 5 | days | | |  |
| 2nd recruitment start | Ag-independent | 93 | days | | |  |
| 2nd recruitment end | Ag-independent | 95 | days | | |  |
| recruitment interval | Ag-independent | 1 | LN ABM time step | | |  |
| recruitment top slice | Ag-independent | 159 | LN ABM grid space | | |  |
| recruitment bottom slice | Ag-independent | 85 | LN ABM grid space | | |  |
| recruitment count | Ag-independent | 1 | count | | |  |
| size | Ag-independent | 2 | LN ABM grid space | | |  |
| max number DCs | Ag-independent | 200 (50-300) | cells | | |  |
| max number DCs resting | Ag-independent | 20 | cells | | |  |
|  |  |  |  | | |  |
| Blood ODE Initial Conditions |  |  |  | | |  |
| naïve CD4+ in BL | Ag-independent | 450 | cells | | | ([Bajaria et al., 2002](#_ENREF_2)) |
| Cognate CD4+ CM in BL | Ag-independent | 0 | cells | | |  |
| Non-cognate CD4+ Effector in BL | Ag-independent | 100 | cells | | |  |
| Non-cognate CD4+CM in BL | Ag-independent | 40 | cells | | |  |
| naïve CD8+ in BL | Ag-independent | 320 | cells | | | ([Roederer et al., 1995](#_ENREF_22)) |
| Cognate CD8+ Effector in BL | Ag-independent | 0 | cells | | |  |
| Cognate CD8+ EM in BL | Ag-independent | 0 | cells | | |  |
| Non-cognate CD8+ Effector in BL | Ag-independent | 100 | cells | | |  |
| Non-cognate CD8+CM in BL | Ag-independent | 40 | cells | | |  |
| Non-cognate CD8+ EM in BL | Ag-independent | 100 | cells | | |  |
| Ag-specific CD4+ CM in BL | Ag-specific | 0 | cells | | |  |
| Ag-specific CD8+ CM in BL | Ag-specific | 0 | cells | | |  |
| Ag-specific CD4+ EM in BL | Ag-specific | 0 | cells | | |  |
| Ag-specific CD8+ EM in BL | Ag-specific | 0 | cells | | |  |
| mean Accum Stim on Ag-specific CD4+ CMs | Ag-specific | 0 | Accumulated stimulation | | | |
| std dev Accum Stim on Ag-specific CD4+CMs | Ag-specific | 0 | Accumulated stimulation | | | |
| mean Accum. Stim on Ag-specific CD8+ CMs | Ag-specific | 0 | Accumulated stimulation | | | |
| mean Accum Stim on Ag-specific CD8+CMs | Ag-specific | 0 | Accumulated stimulation | | | |
| Ag-specific CD4+ EM in NLT | Ag-specific | 0 | cells | | |  |
| Ag-specific CD8+ EM in NLT | Ag-specific | 0 | cells | | |  |
| ODE Parameters |  |  |  | | |  |
| CD4+ Naïve recruitment | Ag-specific | 0.00058 | cells * uL^-1^ * ODE time step^-1^ | | |  |
| CD4+ CM recruitment | Ag-specific | 0.0029 | cells * uL^-1^ * ODE time step^-1^ | | |  |
| CD8+ Naïve recruitment | Ag-specific | 0.000408 | cells * uL^-1^ * ODE time step^-1^ | | |  |
| CD8+ CM recruitment | Ag-specific | 0.00204 | cells * uL^-1^ * ODE time step^-1^ | | |  |
| CD4+ Naïve death rate in blood | Ag-specific | 0.0006 | cells * uL^-1^ * ODE time step^-1^ | | |  |
| CD4+ Effector death rate in blood | Ag-specific | 0.2 | cells * uL^-1^ * ODE time step^-1^ | | |  |
| CD4+ CM death rate in blood | Ag-specific | 0.0017 | cells * uL^-1^ * ODE time step^-1^ | | | ([Homann et al., 2001](#_ENREF_11)) |
| CD4+ EM death rate in blood | Ag-specific | 0.04 | cells * uL^-1^ * ODE time step^-1^ | | | ([Homann et al., 2001](#_ENREF_11)) |
| CD8+ Naïve death rate in blood | Ag-specific | 0.0004 | cells * uL^-1^ * ODE time step^-1^ | | |  |
| CD8+ Effector death rate in blood | Ag-specific | 0.2 | cells * uL^-1^ * ODE time step^-1^ | | |  |
| CD8+ CM death rate in blood | Ag-specific | 0.0001 | cells * uL^-1^ * ODE time step^-1^ | | |  |
| CD8+ EM death rate in blood | Ag-specific | 0.018 | cells * uL^-1^ * ODE time step^-1^ | | | ([Ely et al., 2003](#_ENREF_8)) |
| initial rate of CD4+ Naïve entry to blood from thymus | Ag-specific | 0.272 | cells * uL^-1^ * ODE time step^-1^ | | | ([Bains et al., 2009](#_ENREF_1)) |
| initial rate of CD8+ Naïve entry to blood from thymus | Ag-specific | 0.128 | cells * uL^-1^ * ODE time step^-1^ | | | ([Clark et al., 1999](#_ENREF_6);[Jamieson et al., 1999](#_ENREF_12)) |
| rate of CD4+ Effector egress from blood to peripheral tissue | Ag-specific | 0.02 | cells * uL^-1^ * ODE time step^-1^ | | |  |
| rate of CD4+ EM egress from blood to peripheral tissue | Ag-specific | 0.01 | cells * uL^-1^ * ODE time step^-1^ | | |  |
| rate of CD8+ Effector egress from blood to peripheral tissue | Ag-specific | 0.02 | cells * uL^-1^ * ODE time step^-1^ | | |  |
| rate of CD8+ EM egress from blood to peripheral tissue | Ag-specific | 0.01 | cells * uL^-1^ * ODE time step^-1^ | | |  |
| rate of CD4+ EM conversion to CM in blood | Ag-specific | 0.001 | cells * uL^-1^ * ODE time step^-1^ | | |  |
| rate of CD8+ EM conversion to CM in blood | Ag-specific | 0.0188 | cells * uL^-1^ * ODE time step^-1^ | | | ([Wherry et al., 2003](#_ENREF_24)) |

**Supplementary Table 2. Parameter values for desired memory populations.** Listed here are values of all parameters that were varied from baseline values to achieve the results plotted in Fig 5C.

| **PARAMETER** | **BASELINE** | **I-1** | **I-2** | **I-3** | **II-1** | **II-2** | **II-3** | **III-1** | **III-2** | **III-3** |
| --- | --- | --- | --- | --- | --- | --- | --- | --- | --- | --- |
| *CD4+ T cells* |  |  |  |  |  |  |  |  |  |  |
| max binding time Naïve | 8 | 5 | 3 | 12 | 4 | 6 | 8 | 3 | 4 | 1 |
| max binding time CM | 45 | 49 | 20 | 24 | 53 | 28 | 21 | 58 | 35 | 48 |
| median priming prob | 1000 | 734 | 1116 | 767 | 820 | 912 | 1507 | 1272 | 539 | 973 |
| median effector prob | 2000 | 2417 | 2571 | 2757 | 2119 | 2700 | 2860 | 2514 | 1807 | 2179 |
| extra recruitment | 1.25 | 1.35 | 1.47 | 1.44 | 1.50 | 1.21 | 1.08 | 1.31869 | 1.04 | 1.28 |
| efficiency Central Memory | 6 | 0.65 | 2.45 | 0.70 | 5.47 | 8.07 | 5.13 | 6.17854 | 9.36 | 7.27 |
| prob Effector Memory | 0.1 | 0.06 | 0.30 | 0.12 | 0.36 | 0.36 | 0.38 | 0.178486 | 0.29 | 0.15 |
| Ag-specific binding threshold (a_i_) | 150 | 30 | 90 | 30 |  |  |  |  |  |  |
|  |  |  |  |  |  |  |  |  |  |  |
| *CD8+ T cells* |  |  |  |  |  |  |  |  |  |  |
| max binding time Naïve | 8 | 1 | 9 | 10 | 11 | 9 | 2 | 1 | 4 | 2 |
| max binding time CM | 45 | 24 | 36 | 27 | 54 | 42 | 60 | 45 | 43 | 57 |
| median priming prob | 1000 | 1967 | 1135 | 1337 | 799 | 878 | 896 | 1277 | 1886 | 1475 |
| median effector prob | 2000 | 2124 | 1593 | 1645 | 2628 | 1764 | 2322 | 2519 | 2019 | 1865 |
| extra recruitment | 1.25 | 1.21 | 1.06 | 1.28 | 1.09 | 1.45 | 1.30 | 1.23 | 1.42 | 1.16 |
| efficiency Central Memory | 6.00 | 6.18 | 2.17 | 4.07 | 5.02 | 6.32 | 7.07 | 7.53 | 4.27 | 9.54 |
| prob Effector Memory | 0.10 | 0.19 | 0.26 | 0.16 | 0.13 | 0.23 | 0.14 | 0.21 | 0.32 | 0.38 |
|  |  |  |  |  |  |  |  |  |  |  |
| *Dendritic Cells* |  |  |  |  |  |  |  |  |  |  |
| initial pMHC MDC (Ptot) | 300 | 311 | 403 | 223 | 59 | 342 | 215 | 386 | 170 | 424 |
| prob CD4+ Eff licences MDC | 0.005 | 0.089 | 0.058 | 0.034 | 0.028 | 0.003 | 0.080 | 0.084264 | 0.067 | 0.066 |
|  |  |  |  |  |  |  |  |  |  |  |
| *ODE Parameters* |  |  |  |  |  |  |  |  |  |  |
| EM to CM in blood | 0.001 |  |  |  |  |  |  | 0 | 0 | 0 |
|  |  |  |  |  |  |  |  |  |  |  |
| *# of stimulation events* | 1 | 1 | 2 | 1 | 2 | 3 | 2 | 3 | 3 | 3 |

**Abbreviations**

ABM, agent-based model; LN, lymph node; BL, blood; NLT, non-lymphoid tissue; HEV, high endothelial venule; EL, efferent lymphatics; CM, central memory T cell; EM, effector memory T cell; pMHC, peptide-MHC complex; IDC, immature dendritic cell; MDC, mature dendritic cell; LDC, licensed dendritic cell.

**References**

Bains, I., Antia, R., Callard, R., and Yates, A.J. (2009). Quantifying the development of the peripheral naive CD4+ T-cell pool in humans. *Blood* 113**,** 5480-5487.

Bajaria, S.H., Webb, G., Cloyd, M., and Kirschner, D.E. (2002). Dynamics of naive and memory CD4+ T lymphocytes in HIV-1 disease progression.

Blattman, J.N., Antia, R., Sourdive, D.J., Wang, X., Kaech, S.M., Murali-Krishna, K., Altman, J.D., and Ahmed, R. (2002). Estimating the precursor frequency of naive antigen-specific CD8 T cells. *The Journal of experimental medicine* 195**,** 657-664.

Casrouge, A., Beaudoing, E., Dalle, S., Pannetier, C., Kanellopoulos, J., and Kourilsky, P. (2000). Size estimate of the αβ TCR repertoire of naive mouse splenocytes. *The Journal of Immunology* 164**,** 5782-5787.

Cella, M., Engering, A., Pinet, V., Pieters, J., and Lanzavecchia, A. (1997). Inflammatory stimuli induce accumulation of MHC class II complexes on dendritic cells. *Nature* 388**,** 782-787.

Clark, D.R., de Boer, R.J., Wolthers, K.C., and Miedema, F. (1999). T cell dynamics in HIV-1 infection. *Advances in immunology* 73**,** 301-327.

Demotz, S., Grey, H.M., and Sette, A. (1990). The minimal number of class II MHC-antigen complexes needed for T cell activation. *Science* 249**,** 1028-1030.

Ely, K.H., Roberts, A.D., and Woodland, D.L. (2003). Cutting edge: effector memory CD8+ T cells in the lung airways retain the potential to mediate recall responses. *The Journal of Immunology* 171**,** 3338-3342.

Foulds, K.E., Zenewicz, L.A., Shedlock, D.J., Jiang, J., Troy, A.E., and Shen, H. (2002). Cutting edge: CD4 and CD8 T cells are intrinsically different in their proliferative responses. *The Journal of Immunology* 168**,** 1528-1532.

Henrickson, S.E., Mempel, T.R., Mazo, I.B., Liu, B., Artyomov, M.N., Zheng, H., Peixoto, A., Flynn, M.P., Senman, B., Junt, T., Wong, H.C., Chakraborty, A.K., and von Andrian, U.H. (2008). T cell sensing of antigen dose governs interactive behavior with dendritic cells and sets a threshold for T cell activation. *Nature immunology* 9**,** 282-291.

Homann, D., Teyton, L., and Oldstone, M.B. (2001). Differential regulation of antiviral T-cell immunity results in stable CD8+ but declining CD4+ T-cell memory. *Nature medicine* 7**,** 913-919.

Jamieson, B.D., Douek, D.C., Killian, S., Hultin, L.E., Scripture-Adams, D.D., Giorgi, J.V., Marelli, D., Koup, R.A., and Zack, J.A. (1999). Generation of functional thymocytes in the human adult. *Immunity* 10**,** 569-575.

Kamath, A.T., Henri, S., Battye, F., Tough, D.F., and Shortman, K. (2002). Developmental kinetics and lifespan of dendritic cells in mouse lymphoid organs. *Blood* 100**,** 1734-1741.

Lanzavecchia, A., and Sallusto, F. (2004). Lead and follow: the dance of the dendritic cell and T cell. *Nature immunology* 5**,** 1201-1202.

Linderman, J.J., Riggs, T., Pande, M., Miller, M., Marino, S., and Kirschner, D.E. (2010). Characterizing the dynamics of CD4+ T cell priming within a lymph node. *The Journal of Immunology* 184**,** 2873-2885.

Lindquist, R.L., Shakhar, G., Dudziak, D., Wardemann, H., Eisenreich, T., Dustin, M.L., and Nussenzweig, M.C. (2004). Visualizing dendritic cell networks in vivo. *Nature immunology* 5**,** 1243-1250.

McCune, J.M., Hanley, M.B., Cesar, D., Halvorsen, R., Hoh, R., Schmidt, D., Wieder, E., Deeks, S., Siler, S., and Neese, R. (2000). Factors influencing T-cell turnover in HIV-1–seropositive patients. *Journal of Clinical Investigation* 105**,** R1.

Miller, M.J., Hejazi, A.S., Wei, S.H., Cahalan, M.D., and Parker, I. (2004a). T cell repertoire scanning is promoted by dynamic dendritic cell behavior and random T cell motility in the lymph node. *Proceedings of the National Academy of Sciences of the United States of America* 101**,** 998-1003.

Miller, M.J., Safrina, O., Parker, I., and Cahalan, M.D. (2004b). Imaging the single cell dynamics of CD4+ T cell activation by dendritic cells in lymph nodes. *The Journal of experimental medicine* 200**,** 847-856.

Moon, J.J., Chu, H.H., Pepper, M., McSorley, S.J., Jameson, S.C., Kedl, R.M., and Jenkins, M.K. (2007). Naive CD4+ T Cell Frequency Varies for Different Epitopes and Predicts Repertoire Diversity and Response Magnitude. *Immunity* 27**,** 203-213.

Obar, J.J., Khanna, K.M., and Lefrançois, L. (2008). Endogenous Naive CD8+ T Cell Precursor Frequency Regulates Primary and Memory Responses to Infection. *Immunity* 28**,** 859-869.

Roederer, M., Dubs, J.G., Anderson, M.T., Raju, P.A., Herzenberg, L.A., and Herzenberg, L.A. (1995). CD8 naive T cell counts decrease progressively in HIV-infected adults. *Journal of Clinical Investigation* 95**,** 2061.

Sprent, J., and Tough, D.F. (2001). T cell death and memory. *Science* 293**,** 245-248.

Wherry, E.J., Teichgräber, V., Becker, T.C., Masopust, D., Kaech, S.M., Antia, R., von Andrian, U.H., and Ahmed, R. (2003). Lineage relationship and protective immunity of memory CD8 T cell subsets. *Nature immunology* 4**,** 225-234.
